# Fusion of a rice endogenous *N*-methylpurine DNA glycosylase to a plant adenine base transition editor ABE8e enables A-to-K base editing in rice plants

**DOI:** 10.1007/s42994-024-00138-8

**Published:** 2024-03-21

**Authors:** Yucai Li, Shaoya Li, Chenfei Li, Chen Zhang, Lei Yan, Jingying Li, Yubing He, Yan Guo, Lanqin Xia

**Affiliations:** 1 Institute of Crop Sciences (ICS), Chinese Academy of Agricultural Sciences (CAAS), Beijing, 100081 China; 2 State Key Laboratory of Plant Physiology and Biochemistry, College of Biological Sciences, China Agricultural University, Beijing, 100193 China; 3 Hainan Yazhou Bay Seed Laboratory/National Nanfan Research Institute (Sanya), CAAS, Sanya, 572024 China

**Keywords:** Rice (*Oryza sativa* L), Rice *N*-methylpurine DNA glycosylase (OsMPG), A-to-K base editor (AKBE), Transactivation module VP64

## Abstract

Engineering of a new type of plant base editor for simultaneous adenine transition and transversion within the editing window will greatly expand the scope and potential of base editing in directed evolution and crop improvement. Here, we isolated a rice endogenous hypoxanthine excision protein, N-methylpurine DNA glycosylase (OsMPG), and engineered two plant A-to-K (K = G or T) base editors, rAKBE01 and rAKBE02, for simultaneous adenine transition and transversion base editing in rice by fusing OsMPG or its mutant mOsMPG to a plant adenine transition base editor, ABE8e. We further coupled either OsMPG or mOsMPG with a transactivation factor VP64 to generate rAKBE03 and rAKBE04, respectively. Testing these four rAKBEs, at five endogenous loci in rice protoplasts, indicated that rAKBE03 and rAKBE04 enabled higher levels of A-to-G base transitions when compared to ABE8e and ABE8e-VP64. Furthermore, whereas rAKBE01 only enabled A-to-C/T editing at one endogenous locus, in comparison with rAKBE02 and rAKBE03, rAKBE04 could significantly improve the A-to-C/T base transversion efficiencies by up to 6.57- and 1.75-fold in the rice protoplasts, respectively. Moreover, although no stable lines with A-to-C transversion were induced by rAKBE01 and rAKBE04, rAKBE04 could enable simultaneous A-to-G and A-to-T transition and transversion base editing, at all the five target loci, with the efficiencies of A-to-G transition and A-to-T transversion editing ranging from 70.97 to 92.31% and 1.67 to 4.84% in rice stable lines, respectively. Together, these rAKBEs enable different portfolios of editing products and, thus, now expands the potential of base editing in diverse application scenario for crop improvement.

**Supplementary Information:**

The online version contains supplementary material available at 10.1007/s42994-024-00138-8.

## Introduction

Natural variations between crop cultivars and their wild relatives provide essential genetic resources for crop breeding and improvement (Li et al. 2023a). As a matter of fact, most valuable alleles related to crop yield and other elite agronomic traits are often caused by differences of one or several single-nucleotide polymorphisms (SNPs), or insertion and deletions (indels) with defined lengths (Hu et al. 2015; Jiao et al. 2010; Shang et al. 2022; Wang et al. 2018a, b). However, taking advantages of these SNPs or indels by transferring these alleles into commercialized cultivars, through crossing and back-crossing in conventional breeding, will be very labor intensive and time consuming (Li et al. 2020, 2023a). Generation of valuable alleles of the agriculturally important genes will significantly enrich the crop genetic diversity to meet various demands in breeding practice. The clustered regularly interspaced short palindromic repeat (CRISPR)/CRISPR-associated protein (Cas) (CRISPR/Cas)-mediated base editing has emerged as an efficient, powerful, tool for generating single-nucleotide variations at target sites and directed evolution of agriculturally important genes, significantly facilitating both crop improvement and fundamental research (Chen et al. 2023; Gaudelli et al. 2017; Komor et al. 2016; Kurt et al. 2021; Li et al. 2017, 2023b; Lu and Zhu 2017; Shimatani et al. 2017; Tian et al. 2022; Tong et al. 2023; Yuan et al. 2021; Zhao et al. 2021; Zong et al. 2017). 

At present, cytidine-based editors (CBEs), which are composed of Cas9(D10A) nickase (nCas9), cytidine deaminases from different resources, and uracil-DNA glycosylase inhibitor (UGI), and adenine-based editors (ABEs) composed of nCas9 and adenine deaminases from different resources, enable cytosine (C) to thymine (T) (C-to-T) and adenine (A) to guanine (G) (A-to-G) transitions, respectively (Gaudelli et al. 2017; Komor et al. 2016). CBEs and ABEs have been well developed and widely applied in many plant species (Li et al. 2021, 2023a; Wang et al. 2018a; Xing et al. 2020; Xu et al. 2019, 2021; Yan et al. 2021; Zeng et al. 2020a, b; Zhan et al. 2021; Zhang et al. 2023; Zong et al. 2017). Among them, an optimized ABE8e (TadA8e monomer) substantially increased the efficiencies of A-to-G transition editing in both mammalian cells and rice plants (Richter et al. 2020; Wei et al. 2021). Furthermore, by fusion with the engineered SpCas9 variant SpRY, SpRY-mediated ABE8e further expanded the scope of base editing with highly flexible PAM recognition, but resulted in decreased base editing efficiency in rice (Ren et al. 2021a), and increased off-target effects (Ren et al. 2021b; Wu et al. 2022). CBEs and ABEs enable the directed evolution of agriculturally important genes in crops to generate novel gene/allele resources and germplasm. For example, a CBE- and/or ABE-based gene evolution (BEMGE) strategy was developed to obtain novel allelic variants in *OsALS* and *OsACC* in rice (Kuang et al. 2020; Liu et al. 2020; Wang et al. 2022). 

To enable the generation of multiplex base substitutions, within the editing window of the target site, the dual-base editors for saturated targeted endogenous mutagenesis, STEME and STEME-NG, were developed with the capability of simultaneously performing C-to-T and A-to-G transitions by using a single sgRNA in rice (Li et al. 2020). Later on, STCBE-2 was engineered by fusing an evolved cytidine deaminase, evoFERNY, and TadA8e to nCas9-NG to further improve the dual-base editing efficiency and expand the editing scope. STCBE-2 outperformed STEME-NG in conducting simultaneous C-to-T and A-to-G base editing by 2.9- and 13.2-fold, respectively (Zhang et al. 2023). Besides, a new base editor, GBE (glycosylase-based editor), composed of a cytidine deaminase fused to nCas9 and an uracil-DNA glycosylase (UNG), has been developed to convert cytosine (C) to guanine (G) (Kurt et al. 2021; Zhao et al. 2021). The GBE system further expanded the base editor toolbox and was successfully applied in rice, although the efficiencies of C-to-G in plants were very low (Tian et al. 2022). However, as some elite agronomic traits are caused by A-to-C/T transversions, in a specific target gene (Hua et al. 2022; Ma et al. 2015; Zhao et al. 2018), development of base editing tools enabling A-to-T and/or A-to-C base transversions will greatly expand the potential for base editing in both directed evolution through saturated mutagenesis and crop improvement.

Recently, a new type of base editor, AYBE, has been developed for A-to-Y (Y = C/T) editing, by fusing an adenine base editor with the human hypoxanthine excision protein *N*-methylpurine DNA glycosylase (hMPG) in mammalian cells (Tong et al. 2023). Later on, two adenine transversion editors, AXBEs and ACBEs, for efficient A-to-C base editing, were developed by fusing TadA8e with a mouse hypoxanthine excision protein *N*-methylpurine DNA glycosylase (mMPG or mAAG) (Chen et al. 2023), indicating the MPG derived from different resources may have different base excision repair activities. Interestingly, the plant adenine base editors, pAKBEs, engineered by fusions of either the rice codon-optimized hMPG or its mutant mhMPG to plant ABE8e or ABE-TadA9 only generated stable lines with A-to-G/T base editing in rice plants, with no detected or very few lines with A-to-C transversion editing (Li et al. 2023b; Wu et al. 2023). Thus, exploring the MPGs from different resources may benefit the development of different adenine base editors for various  applications. Like mammalian cells, rice also has MPG ortholog (Fig. S1). However, the function of this rice MPG (OsMPG) has not been elucidated. Whether OsMPG could be employed to develop an efficient plant adenine base editor for precise A-to-G/C/T editing in plants remains to be established.

In this study, we isolated a rice endogenous OsMPG and engineered two plant A-to-K (K = G or T) base editors, rAKBE01 and rAKBE02, for simultaneous precise adenine base transition and transversion editing in rice, by fusing OsMPG or its mutant mOsMPG to a plant adenine transition base editor, ABE8e. We further coupled either OsMPG or its mutant mOsMPG with a transactivation factor, VP64, to generate rAKBE03 and rAKBE04, respectively. We then investigated the performances of these rAKBEs, both in rice protoplast and stable lines. We demonstrated that these rAKBEs enabled A-to-K base editing in rice. Among which, rAKBE04 exhibited the best performance with an expanded editing window, enabling increased A-to-G transition editing, along with decreased A-to-T transversion and the occurrences of fewer indels within the editing window of targets, in comparison to our previously reported hMPG-based plant A-to-K base editors, pAKBEs (Li et al. 2023b). Development of base editors with different editing profiles will assist in generating alleles with desired base substitutions, in different contexts, for precise crop breeding as well as creating new germplasm through directed evolution.

**Fig. 1:**
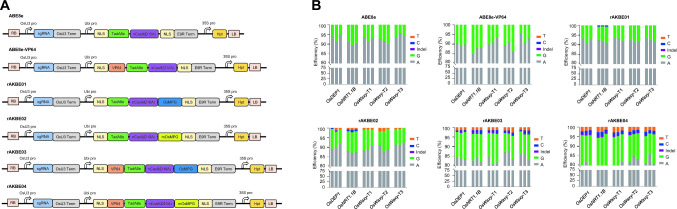
Schematic diagrams of four rAKBEs and their performances in rice protoplasts. **A** Schematic diagrams of four rAKBEs. pABE8e, ABE8e-VP64, original rAKBE01 and three optimized rAKBEs for A-to-K base editing. rAKBE01, version 1 of rAKBE with wild-type OsMPG; rAKBE02, rAKBE with mutated OsMPG (mOsMPG); rAKBE03, rAKBE with wild type OsMPG and VP64; rAKBE04, rAKBE with mOsMPG and VP64. In these rAKBEs, the rAKBEs are driven by a maize *Ubiquitin *promotor (*Ubi*), whereas the gRNA is expressed under the control of an *OsU3 *promoter and terminated with ‘TTTTTTT’, respectively. mOsMPG, OsMPG including the G162R/N168S mutations; VP64, four copies of VP16 transactivation domain. **B** The performance of rAKBEs in rice protoplasts. Bar plots showing the on-target DNA base editing frequencies with ABE8e, ABE8e-VP64 and rAKBEs, using five endogenous targets in rice protoplast, respectively. The frequencies of targeted A-to-T and A-to-C are highlighted in orange and blue, respectively. The frequencies of targeted A-to-G is highlighted in green. The frequencies of indels that occurred in the protospacer region are shown in purple. The editing outcomes of each target, by different rAKBEs, are calculated based on three independent biological replicates (each replicate was performed with 3 repeats), and are displayed side-by-side

## Results

### The rAKBEs enable A-to-G/C/T transition and transversion base editing in rice protoplasts

We first identified a hMPG ortholog, OsMPG (LOC_Os02g53430), in a *japonica* rice *cv* Zhonghua 11 by using the amino acid sequences of hMPG to conduct BLAST searches against the databases of NCBI (https://www.ncbi.nlm.nih.gov) (Fig. S1-A). Furthermore, based on the fact that the hMPG mutant containing seven mutations (G163R, N169S, S198A, K202A, G203A, S206A, K210A) could significantly improve the efficiency of adenine base conversion in mammalian cell line (Tong et al. 2023) and the mutations of S198A, K202A, G203A, S206A, K210A in mhMPG are presented in OsMPG, we further mutated the remaining two amino acids, G162R and N168S, in the conserved region of amino acid sequence of OsMPG to generate mOsMPG (Fig. S1-B). The sequence alignments of these hMPG, mhMPG, OsMPG, and mOsMPG are illustrated in Fig. S1C. The homologies between hMPG/mhMPG and OsMPG/mMPG are very low, with an amino acid sequence identity of only 27.36% between hMPG and OsMPG (Fig. S1-C). We then engineered two adenine transition and transversion base editors (rAKBEs), rAKBE01 and rAKBE02, by fusing either the OsMPG or its mutant version mOsMPG (G162R, N168S) to the C-terminus of an adenine transition base editor, ABE8e, with a 13-amino acid linker (Fig. 1A and Fig. S1). Furthermore, given that VP64, a transactivation module composed of four copies of VP16 transactivation domain, could facilitate remodeling and unfolding of condensed chromatin and, thus, accessibility of CGBE to target sites, resulting in improved C-to-G base editing efficiency (Dong et al. 2022; Li et al. 2023b; Tumbar et al. 1999), we further fused the VP64 to the N-terminal of TadA8e in rAKBE01 and rAKBE02, to generate rAKBE03 and rAKBE-04, respectively (Fig. 1A). We also fused VP64 to ABE8e to generate ABE8e-VP64, as a control (Fig. 1A).

**Fig. 2:**
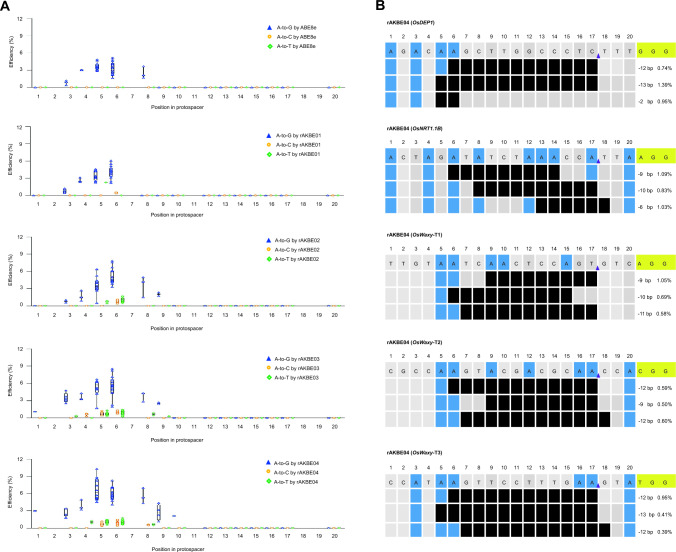
The editing windows and on-target indels induced by rAKBEs in rice protoplasts. **A** The editing windows of rAKBEs. The X-axis represents the protospacer positions 1-20 bases in the target region, counting PAM as positions 21-23. Single dots represent individual biological samples (each replicate with 3 repeats). In total, 3 independent biological replicates were performed, per site. Horizontal lines indicate the median; whiskers extend to minimum and maximum efficiencies. All values are presented as mean ± SEM of three repeats. **B** The on-target indels induced by rAKBEs. The small indels generated from the 5′-deaminated ‘A’ base to the nCas9 nicking site, or betweet two ‘A’ bases in the protospacer regions, by rAKBEs. The proportions of indels versus total reads are shown below PAM. PAMs are highlighted in yellow, and black bars represent the deleted nucleotide fragment. “–”, deletion

We then investigated the adenine base editing activities of ABE8e, ABE8e-VP64, and these four rAKBEs in rice protoplasts by targeting endogenous *OsDEP1*, *OsNRT1.1B*, *OsWaxy*-T1, *OsWaxy*-T2, and *OsWaxy*-T3, respectively (Tables S1 and S2). The same gRNA expression cassettes were cloned into ABE8e, ABE8e-VP64, and each of the four rAKBEs, respectively, and then transiently expressed in rice protoplasts. The targeted fragments of these selected target genes were amplified by PCR, then processed for Hi-Tom high-throughput sequencing (Liu et al. 2019). As expected, ABE8e induced A-to-G transition, at average efficiencies of 5.44–9.49%, with a typical ABE8e editing window of A3–A8, counting the PAM position as 21–23 (Figs. 1B, 2A and Table S3). In comparison with ABE8e, ABE8e-VP64 could enhance the A-to-G base transversion efficiencies by up to 1.47-fold (9.33%/6.33% at *OsWaxy*-T3). Of the five tested endogenous targets, the rAKBE01 could induce A-to-G base transitions at positions A3–A6 in the protospacer region, at average frequencies of 7.00–8.15%, which were similar to the ABE8e (Figs. 1B and 2A and Table S3). Whereas rAKBE01 only enabled A-to-C transversion at *OsNRT1.1B* targets, with average editing efficiencies of 0.47% at protospacer position A6 (Figs. 1B, 2A and Table S4). As expected, rAKBE02 with mOsMPG enabled A-to-G base transitions, at average efficiencies of 7.89–10.76%, with an editing window of A3–A9 (Figs. 1B, 2A and Table S3). In comparison to rAKBE01, rAKBE02 enhanced the A-to-G base transitions efficiencies by up to 1.41-fold (11.08%/7.87% at *OsWaxy*-T1). It also induced the A-to-C base editing efficiencies by 1.12% and 0.64% at *OsNRT1.1B* and *OsDEP1* target loci, respectively (Figs. 1B and 2A and Table S4), which significantly improved the A-to-C base transversion by up to 2.07-fold (1.12%/0.54% at *OsNRT1.1B*). The rAKBE02 also enabled A-to-T transversion at *OsDEP1*, *OsNRT1.1B*, *OsWaxy*-T1, *OsWaxy*-T2, and *OsWaxy*-T3 targets, with average editing efficiencies of 0.97%, 0.85%, 0.72%, 1.21%, and 0.54%, respectively (Fig. 1B and Table S4). As for the editing window, rAKBE02 had a wider editing window of A3–A9 (Fig. 2A). 

By coupling with VP64, rAKBE03 enabled higher levels of A-to-G transitions across all five targets with a further expanded editing window of A1–A9, with the average editing efficiencies reached up to 13.65%, 13.49%, 14.38%, 10.58%, and 11.72% at *OsDEP1*, *OsNRT1.1B*, *OsWaxy*-T1, *OsWaxy*-T2, and *OsWaxy*-T3 targets, respectively (Fig. 1B, 2A and Table S3). Compared with rAKBE02, rAKBE03 enhanced the A-to-G base transitions efficiencies by up to 1.61-fold (13.18%/8.21% at *OsWaxy*-T3) (Fig. 1B and Table S3). As shown in Fig. 1B and Table S4, rAKBE03 also enabled the A-to-C and A-to-T base transversion, at average frequencies of 0.89% and 1.48% at *OsDEP1*, 1.23% and 1.41% at *OsNRT1.1B*, 1.08% and 1.45% at *OsWaxy*-T1, 1.12% and 1.99% at *OsWaxy*-T2, and 1.07% and 1.54% at *OsWaxy*-T3, respectively. The average A-to-Y transversion efficiencies were 2.36% at *OsDEP1*, 2.64% at *OsNRT1.1B*, 2.53% at *OsWaxy*-T1, 3.11% at *OsWaxy*-T2, and 2.61% at *OsWaxy*-T3, respectively (Table S4). In comparison with rAKBE02, rAKBE03 not only increased the A-to-Y transversion efficiencies by 1.29-fold (2.40%/1.86% at *OsDEP1*) to 4.93-fold (2.96%/0.60% at *OsWaxy*-T3), but also enabled A-to-C transversion at the non-editable sites, such as *OsWaxy*-T1, *OsWaxy*-T2, and *OsWaxy*-T3 (Fig. 1B and Table S4). The rAKBE03 enabled A-to-G transition, and A-to-C and A-to-T base transversions at positions A1–A9 (Fig. 2A). This editing result indicated that coupling with VP64 might facilitate chromosome unfolding and accessibility of rAKBE to the nearby ‘A’ bases in the protospacer region. 

As expected, rAKBE04, coupled with mOsMPG and VP64, enabled an even higher A-to-G base transitions, at average efficiencies of 13.19–16.52%, with a more expanded editing window of A1–A10 (Figs. 1B, 2A and Table S3). In comparison with ABE8e, ABE8e-VP64, rAKBE01, rAKBE-02, and rAKBE03, rAKBE04 could enhance the A-to-G base transversion efficiencies by up to 2.46-fold (15.55%/6.33% at *OsWaxy*-T3), 1.67-fold (15.55%/9.33% at *OsWaxy*-T3), 2.11-fold (16.58%/7.87% at *OsNRT1.1B*), 1.89-fold (15.55%/8.21% at *OsWaxy*-T3), and 1.18-fold (15.55%/13.18% at *OsWaxy*-T3), respectively, with the highest A-to-G editing efficiency reached being up to 17.87% at the *OsNRT1.1B* target site (Table S3). Furthermore, compared with the pAKBEv4-mhMPG-VP64 in our previous report (Li et al. 2023b), rAKBE04 exhibited higher editing efficiency of A-to-G by up to 3.06-fold (15.55%/5.09% at *OsWaxy*-T3) (Fig. 1B and Table S3). The rAKBE04 further improved the A-to-C editing efficiencies in rice protoplast, across the five target loci. It enabled A-to-C transversion at *OsDEP1*, *OsNRT1.1B*, *OsWaxy*-T1, *OsWaxy*-T2, and *OsWaxy*-T3 targets, with average editing efficiencies of 1.30%, 1.65%, 1.33%, 1.24%, and 1.41%, respectively (Fig. 1B and Table S4). 

In comparison with rAKBE03, rAKBE04 enhanced the A-to-C base transversion efficiencies by up to 1.61-fold (1.59%/0.99%), 1.20-fold (1.96%/1.63%), 1.29-fold (1.60%/1.24%), 1.02-fold (1.36%/1.33%), and 1.19-fold (1.52%/1.28%) at *OsDEP1*, *OsNRT1.1B*, *OsNRT1.1B*, *OsWaxy*-T1, *OsWaxy*-T2, and *OsWaxy*-T3 targets, respectively, with the highest efficiency of A-to-C transversion reached up to 1.96% at *OsNRT1.1B* in rice protoplasts (Fig. 1B and Table S4). In addition, the rAKBE04 enabled A-to-T transversion at *OsDEP1*, *OsNRT1.1B*, *OsWaxy*-T1, *OsWaxy*-T2, and *OsWaxy*-T3 targets with average editing efficiencies of 1.97%, 2.22%, 2.51%, 1.85%, and 2.03% at positions A4, A5, A6, and A8, respectively (Fig. 2A). In comparison with rAKBE02, rAKBE04 improved the A-to-T base transversion efficiencies at all five targets, and the editing efficiency was improved by up to 4.03-fold (2.42%/0.60%) at *OsWaxy*-T3 target (Table S4). In comparison with rAKBE03, rAKBE04 also improved the A-to-T base editing efficiencies by 1.42-fold (2.21%/1.56%), 1.82-fold (2.66%/1.46%), 1.94-fold (2.95%/1.52%), and 1.32-fold (2.42%/1.83%) at *OsDEP1*, *OsNRT1.1B*, *OsWaxy*-T2, and *OsWaxy*-T3 targets, respectively (Table S4). It is noteworthy that, in comparison with ABE8e, ABE8e-VP64, and the other rAKBEs, rAKBE04 exhibited a wider editing window of A1–A10 (Fig. 2A). Collectively, these results indicate that rAKBE04 outperforms other rAKBEs in expanding the editing window and enables more efficient A-to-G/C/T in rice protoplasts.

### The rAKBEs generated on-target indels in rice protoplasts

Because MPGs are involved in the base excision repair (BER) pathway, the excision of deoxyinosine (I) by MPGs in the non-targeted DNA strand usually results in lesions along with the process of adenine base editing (Chen et al. 2023; Li et al. 2023b; Tong et al. 2023). As expected, we observed the occurrences of on-target indels induced by rAKBE03 and rAKBE04 in rice protoplasts (Fig. 2B and Table S5). Deep sequencing results suggested that most of these on-target indels occurred precisely between the nicks produced by nCas9 (D10A) and ‘A’ base, or between two ‘A’ bases in the protospacer region (Fig. 2B), for example, a 12 bp deletion between A6 and the nick of nCas9 (D10A), a 13 bp deletion between A5 and the nick of nCas9 (D10A), and 2 bp deletion between positions A5 and A6 at *OsDEP1* target (Fig. 2B). We also detected the occurrences of a 10 bp deletion between the nick of nCas9 (D10A) and A8, a 9 bp deletion between A6 and A14, and a 6 bp deletion between A13 and T18 in the protospacer region at *OsNRT1.1B,* with the average frequencies of 0.83%, 1.09%, and 1.03%, respectively (Fig. 2B). Besides, a 9 bp deletion between A9 and the nCas9 (D10A) nick, a 10 bp deletion between A6 and A15, and an 11 bp deletion between the nick of nCas9 (D10A) and T7 were observed, at frequencies of 1.05%, 0.69%, and 0.58% at *OsWaxy-*T1, respectively (Fig. 2B). Furthermore, a 12 bp deletion between A6 and nCas9 (D10A) nick, a 9 bp deletion between A9 and nCas9 (D10A) nick, and a 12 bp deletion between G7 and C18 were generated, at frequencies of 0.59%, 0.50%, and 0.60% at *OsWaxy-*T2, respectively (Fig. 2B). Moreover, a 12 bp deletion between A6 and nCas9 (D10A) nick, a 13 bp deletion between A5 and nCas9 (D10A) nick, and a 12 bp deletion between G7 and G18 were generated, at frequencies of 0.95%, 0.41%, and 0.39% at *OsWaxy-*T3, respectively (Fig. 2B). Generation of on-target indels, with a defined length, offers benefits for the functional characterization of *cis*- and *trans*-elements in the promoter region of target genes of interest.

### Performance of rAKBEs in rice stable lines

**Fig. 3:**
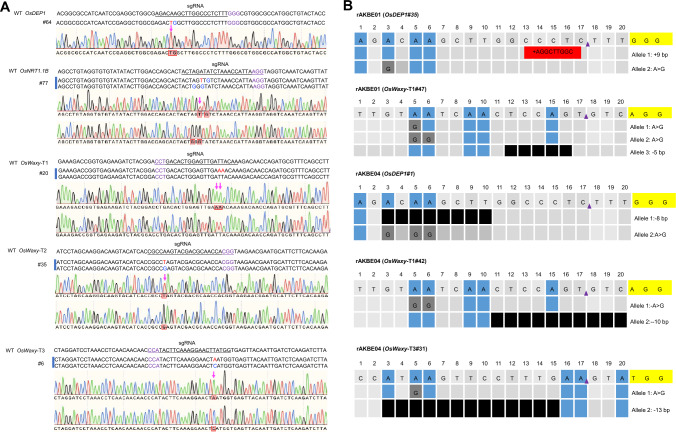
Representative editing profiles of each target induced by rAKBE01 and rAKBE04 in rice stable lines. **A** Representative Sanger sequencing chromatograms of five target loci. *OsDEP1*, *OsNRT1.1B*, *OsWaxy*-T1, *OsWaxy*-T2 and *OsWaxy*-T3 target sites showing desired A-to-T transversion editing. A-to-T transversion shown in red, and A-to-G transitions shown in blue. The sgRNA target sequences are underlined and their PAM sites are in purple. **B** Representative small indels generated by rAKBE01 and rAKBE04 in rice stable lines in the T0 generation. PAMs are highlighted in yellow, and black bars represent the deleted nucleotide fragment. Red bars represent the inserted nucleotides. “–”, deletion; “+”, insertion.

To further investigate whether rAKBEs can induce A-to-G/T/C base editing, in the rice stable lines, we evaluated the base editing outcomes of rAKBE01 and rAKBE04 at *OsDEP1*, *OsNRT1.1B*, *OsWaxy*-T1, *OsWaxy*-T2, and *OsWaxy*-T3 targets, respectively. Genotyping the independent rice lines indicated that the dominant occurrences of A-to-G transitions was detected at all the tested five target sites, at frequencies ranging from 70.59 to 98.70% in the tested stable lines (Table 1). The A-to-T transversions were successfully generated by rAKBE01 at *OsNRT1.1B* and *OsWaxy*-T3 target sites, with efficiencies of 1.30% (1/77) and 1.47% (1/68), respectively (Fig. 3A, Table 1 and Fig. S3). The genotypes of these rice stable lines are indicated in Fig. 3A, Table 2, and Fig. S3. For the *OsNRT1.1B* target site, line #77 carried one A-to-T transversions at protospacer position A6 and three A-to-G transition at protospacer positions A4, A6, and A8 (Fig. 3A, Table 2). For the *OsWaxy*-T3 target site, line #4 carried one A-to-T transversion, at protospacer position A5, and one A-to-G transition at protospacer position A5 (Table 2). Notably, similar to our results in rice protoplast, the rAKBE04 outperformed rAKBE01 in inducing A-to-T transversion editing in rice stable lines (Table 1). We achieved the A-to-T transversion editing efficiencies of 3.85% (3/78), 2.33% (1/43), 1.67% (1/60), 2.60% (2/77), and 4.84% (3/62) at *OsDEP1*, *OsNRT1.1B*, *OsWaxy*-T1, *OsWaxy*-T2, and *OsWaxy*-T3 targets in rice stable lines, respectively, with the highest A-to-T transversion editing efficiency reached up to 6.35% at *OsWaxy*-T3 (Table 1). 

In comparison with rAKBE01, the rAKBE04 enabled A-to-T base editing at non-editable targets, such as *OsDEP1, OsWaxy*-T1, and *OsWaxy*-T2, and increased the A-to-T transversion editing efficiencies by 1.75- and 3.29-fold at *OsNRT1.1B* (2.33%/1.30%) and *OsWaxy*-T3 (4.84%/1.47%) in rice stable lines, respectively (Tables 1, 2, and Fig. S4). Moreover, we obtained 7 independent lines with both A-to-T transversions and A-to-G transition at *OsWaxy*-T1, *OsWaxy*-T2, and *OsWaxy*-T3 targets, respectively (Tables 1, 2, and Fig. S4). Importantly, the on-target indels occurred at frequencies of 5.13%, 2.33%, 1.67%, 1.30%, and 4.84% at *OsDEP1*, *OsNRT1.1B*, *OsWaxy*-T1, *OsWaxy*-T2, and *OsWaxy*-T3 targets in these edited stable lines, respectively (Fig. S5, Table 1), significantly lower than the frequencies of indels induced by the hMPG-based pAKBEs, which were 34.15%, 5.56%, 14.08%, 25.61%, and 10.34% at *OsDEP1*, *OsNRT1.1B*, *OsWaxy*-T1, *OsWaxy*-T2, and *OsWaxy*-T3 targets, respectively (Li et al. 2023b). It is noteworthy that the relatively lower frequencies of on-target indels, induced by the rAKBEs, may represent an advantage for directed evolution of agriculturally important genes in which on-target indels, within the target sequences that encode key functional domains, are lethal or highly undesirable.

**Table 1:** The editing outcomes of rAKBE01 and rAKBE04 at different endogenous loci in rice stable lines

Target sites	Base editor	No. of independent transgenic lines	No. of independent edited lines	A-to-G editing efficiency	A-to-T editing efficiency	Indel efficiency
*OsDEP1*						
	rAKBE01	77	76	98.70% (76/77)	0.00% (0/77)	1.30% (1/77)
	rAKBE04	78	72	92.31% (72/78)	3.85% (3/78)	5.13% (4/78)
*OsNRT1.1b*						
	rAKBE01	77	68	88.31% (68/77)	1.30% (1/77)	0.00% (0/77)
	rAKBE04	43	31	72.09% (31/43)	2.33% (1/43)	2.33% (1/43)
*OsWaxy-T1*						
	rAKBE01	60	53	88.33% (53/60)	0.00% (0/60)	3.33% (2/60)
	rAKBE04	60	54	90.00% (54/60)	1.67% (1/60)	1.67% (1/60)
*OsWaxy-T2*						
	rAKBE01	34	32	94.12% (32/34)	0.00% (0/34)	0.00% (0/34)
	rAKBE04	77	71	92.21% (71/77)	2.60% (2/77)	1.30% (1/77)
*OsWaxy-T3*	rAKBE01	68	48	70.59% (48/68)	1.47% (1/68)	2.94% (2/68)
	rAKBE04	62	43	70.97% (44/62)	4.84% (3/62)	4.84% (3/62)

**Table 2:** Representative genotypes of rice lines with A-to-K produced by rAKBE01 and rAKBE04

Base editors	Targets	Lines in T= generation	Genotype		Positions in protopspacer region
rAKBE01	NRT1.1B	#77	Bi	Allele 1: A6 > T6, A8 > G8 Allele 2: A4 > G4, A6 > G6	6
	OsWaxy-T3	#4	Bi	Allele 1: T5 > A5 Allele 2: T5 > C5	5
rAKBE04	OsDEP1	#64	Ho	A5 > T5, A6 > G6	5
		#32	Chi	Allele 1: A5 > T5, A3 > G3 Allele 2: A3 > G3 Allele 3: A3 > G3, A5 > G5 Allele 4: A5 > G5, A6 > G6 Allele 5: A3 > G3, A5 > G5, A6 > G6	5
		#72	Bi	Allele 1: A5 > T5, A3 > G3, A6 > G6 Allele 2: A3 > G3, A5 > G5	5
	OsNRT1.1B	#43	Chi	Allele 1: A6 > T6, A8 > G8 Allele 2: A4 > G4, A6 > G6 Allele 3: A4 > G4, A8 > G8	6
	OsWaxy-T1	#20	He	Allele 1: T5 > A5, T6 > A6; Allele 2: WT	5, 6
	OsWaxy-T2	#35	Bi	Allele 1: A5 > T5 Allele 2: A6 > G6	5
		#77	Bi	Allele 1: A5 > T5 Allele 2: A9 > G9	5
	OsWaxy-T3	#6	Bi	Allele 1: T5 > A5 Allele 2: T5 > C5	5
		#51, #56	He	Allele 1: T5 > A5 Allele 2: WT	5
		#17	He	Allele 1: T5 > A5 Allele 2: T5 > C5 Allele 3: T3 > C3, T5 > C5	5

PAM sequence is underlined in each target. The number of individual plants included in each independent line is shown in bracket. In the genotype column, A-to-G or T-to-C transitions are shown in black, whereas A-to-T or T-to-A transversions are shown in red. Ho, homozygous line; He, heterozygous lines; Bi, biallelic line; Chi, Chimeric line

Modulating chromatin accessibility, by fusion of a synthetic transcription activation domain (TV) to Cas9, enhanced the CRISPR/Cas9-mediated genome editing efficiency in rice (Liu et al. 2019). Our study reinforces the fact that coupling VP64 with rAKBEs facilitates their editing activities (Fig. 1B and Table 1). To test whether VP64 had any negative effects, such as enhanced expression of target genes, we further examined the expression levels of *OsDEP1* and *OsNRT1.1B* in edited stable lines by RT-qPCR (quantitative reverse transcription PCR) analyses. For rAKBE01 and rAKBE04, three independent lines with either A-to-Y or A-to-G base editing obtained at *OsDEP1* and *OsNRT1.1B* were selected for RT-qPCR analyses, respectively. As shown in Fig. S6, the RT-qPCR results indicated that there were no significant differences in the expression levels of *OsDEP1* and *OsNRT1.1B,* between wild-type control and the edited lines generated by these two rAKBEs. This result suggested that VP64 had no collateral effects on the expression of the targeted genes, at least in these tested target genes and the edited stable lines.

## Discussion

Development of plant AKBEs enabling simultaneous A-to-G, A-to-T and on-target indels would be highly beneficial for developing novel alleles by directed evolution of agriculturally important genes during crop improvement. In this study, we isolated a rice endogenous *OsMPG* which has not been previously functionally characterized. We engineered four rAKBEs, through fusions of OsMPG, mOsMPG, or/and VP64 with ABE8e, respectively (Fig. 1A). Investigating the base editing activities of these four rAKBEs, in rice protoplasts, demonstrated that rAKBE04 showed the best performance and could generate more comprehensive editing outcomes, including A-to-G, A-to-C, A-to-T, and on-target indels across the five tested target sites in the rice protoplasts (Fig. 1B). Furthermore, whereas rAKBE04 increased the A-to-C/T base transversion editing efficiencies, across all the five target sites, compared to the other rAKBEs (rAKBE01, rAKBE02, and rAKBE03), it also substantially improved the A-to-G base editing efficiencies, compared to ABE8e and ABE8e-VP64, as well as our previously developed mhMPG-based pAKBEs (Li et al. 2023b) in a parallel experiment in rice protoplasts (Fig. 1B). In addition, rAKBE04 expanded the editing window from A3–A6 to A1–A10 compared with rAKBE01 (Fig. 2A). Furthermore, we examined the off-target possibility for each on-target site, and no off-target effects were detected at the potential off-target sites (CRISPR-GE, http://skl.scau.edu.cn/) in the tested lines (Table S6), indicating the on-target specificities of rAKBE04 in rice stable lines. These results suggested that rAKBE04 can serve as a useful alternative for directed evolution of agricultural important genes. Moreover, as shown in Figs. 1B and 2A, coupling with VP64 indeed could facilitate the accessibility of the CRISPR complex and, thus, enhance the base editing efficiencies, and the mOsMPG and VP64 combination may have synergic effect on boosting the adenine transition and transversion base editing efficiency, consistent with previous reports (Campos and Reinberg 2009; Dong et al. 2022; Li et al. 2023b).

In rice stable lines, as demonstrated in Table 1 and Fig. S5, rAKBE04 induced higher efficacies of A-to-G transition editing, decreased A-to-T transversion base editing, and lowered on-target indel efficiencies at the same five target sites in comparison to the previously reported hMPG-based pAKBEs (Li et al. 2023b). In comparison with the performances of hMPG (Tong et al. 2023) and mMPG (Chen et al. 2023), in mammalian cells, the A-to-C and A-to-T transversion base editing efficiencies mediated by the hMPG-based pAKBEs are also relative lower or even undetectable for A-to-C transversion in rice stable lines (Li et al. 2023b; Wu et al. 2023). This result suggests that rice may have unique and unidentified BER mechanisms to be explored. Furthermore, similar to our previous report (Li et al. 2023b), no lines with A-to-C transversion were detected in the tested stable lines, whereas comparable A-to-C and A-to-T transversions were induced by rAKBEs, across five target loci in rice protoplasts (Fig. 1B). This phenomenon may be due to the following reasons. For rAKBEs and the previously reported pAKBEs (Li et al. 2023b; Wu et al. 2023), similar to AYBE or ACBE developed in mammalian cells (Chen et al. 2023; Tong et al. 2023), the adenine (A) in the non-targeting strand is deaminated by ABE8e and converted into deoxyinosine (I) and then excised by hMPG/mhMPG/mMPG (Chen et al. 2023; Tong et al. 2023) or OsMPG/mOsMPG. The excision of deoxyinosine then induces the generation of apurinic/apyrimidinic (AP) sites, in the non-targeting strand. The AP sites are then hydrolyzed by AP lyase, inside the cells, resulting in a lesion in the non-targeting strand. Versatile base editing outcomes including A-to-G/C/T and indels could be generated following triggered translesion synthesis (TLS) over AP sites in the BER pathway or DNA replication (Li et al. 2023b; Tong et al. 2023). One of the editing products, the A-to-C base transversion, may act as a target of the endogenous uracil-DNA *N*-glycosylase (UNG) in rice, via the BER pathway (Tian et al. 2022), thus resulting in either C-to-G or C-to-A transversions. Indeed, according to this principle, GBE base editors have been developed both in mammalian cells and plants (Kurt et al. 2021; Tian et al. 2022; Zhao et al. 2021). Surprisingly, the GBE base editor developed for plant C-to-G transversion is not as efficient as its counterpart in mammalian cells (Kurt et al. 2021; Tian et al. 2022; Zhao et al. 2021). These results indicate that the differences in genetic backgrounds, between plants and mammalian cells, may affect the BER activities of these base editors, although the performances of both rAKBEs and pAKBEs remain to be investigated in other plant species. In addition, given that both the rAKBEs in this study and the pAYBEs developed previously (Li et al. 2023b; Wu et al. 2023) were transiently expressed in rice protoplast, but constitutively expressed in rice stable lines, we speculate that the transient A-to-C base transversion could not be processed efficiently by the endogenous UNG, in rice protoplast, thus leading to the observed results, where comparable A-to-C and A-to-T transversion editing efficiencies were detected in rice protoplasts, but no or fewer lines with A-to-C transversion were detected in rice stable lines. The potential mechanism underlying this phenomenon remains to be investigated in the future. Thus, manipulating the BER pathway, DNA replication, or exploiting novel alkyladenine DNA glycosylases, will be necessary in order to improve A-to-C/T, especially A-to-C, transversion base editing efficacy in plants.

Nevertheless, we here demonstrate that a rice OsMPG-based rAKBEs enable A-to-K base editing in rice plants. The rAKBE04 enables higher levels of A-to-G transition editing, decreased A-to-T transversion editing, and lower occurrences of on-target indels in A-to-K base editing in rice stable lines. This characteristic of rAKBE04 may serve as a valuable toolkit as complementation to the current repertoire of base editors for both directed evolution of agriculturally important genes and crop improvement.

## Methods and materials

### Construction of the A-to-K base editing vectors

The pHUE411-NLS-TadA8e-Linker(32aa)-nCas9(D10A)-NLS-E9T vector (hereafter referred to as the ABE8e vector original) used in the study was constructed by fusing TadA8e-Linker (32aa) to the N-Terminal of nCas9(D10A) in vector pHUE411-NLS-Cas9-NLS-E9t (Xing et al. 2014), which placed TadA8e-Linker(32aa)-nCas9(D10A) under the control of the maize (*Zea mays*) ubiquitin gene promoter. An engineered *OsMPG* (rice N-methylpurine DNA glycosylase protein, also known as alkyl adenine DNA glycosylase, AAG) was synthesized commercially (TSINGKE, Beijing, China). For rAKBE01, the *OsMPG* fragment was cloned into the ABE8e vector to generate pHUE411-ZmUbi-NLS-TadA8e-Linker(32aa)-nCas9(D10A)-linker(13aa)-OsMPG- NLS-E9t. Mutated *OsMPG* including the G162R and N168S mutations was introduced to replace the *OsMPG* fragment in rAKBE01 to generate the vector pHUE411-ZmUbi-NLS-TadA8e-Linker(32aa)-nCas9(D10A)-linker(13aa)-mhMPG-NLS-E9t (referred to as rAKBE02 in this study). For rAKBE03, the transactivation module Vp64 (Dong et al. 2022) was rice codon-optimized and synthesized commercially (GENEWIZ, Tianjin, China). NLS-VP64-TadA8e-Linker(32aa)-nCas9 fragment was amplified by overlapping extension PCR using corresponding primers, and then cloned into *Avr*II-*Sbf*I digested rAKBE01 to generate pHUE411-ZmUbi-NLS-VP64-linker(13aa)-TadA8e-Linker(32aa)-nCas9(D10A)-linker(13aa)-hMPG-NLS-E9t (referred to as rAKBE03 in this study). The vector pHUE411-ZmUbi-NLS-VP64-linker (13aa)-TadA8e-Linker (32aa)-nCas9 (D10A)-linker(13aa)-mhMPG-NLS-E9t (referred to as rAKBE04 in this study) was obtained by introducing the VP64 into rAKBE03. The gRNA expression cassette under the control of the *OsU3* promoter was inserted into the *Hin*d III digested rAKBE01, rAKBE02, rAKBE03, and rAKBE04, respectively. The double strands of protospacers were synthesized, annealed, and inserted into *Bsa*I digested gRNA expression cassette in rAKBE01, rAKBE02, rAKBE03, and rAKBE04, individually. All vectors were confirmed by Sanger sequencing (TSINGKE, Beijing, China). A One-Step Cloning Kit (ClonExpress II One-Step Cloning Kit, Vazyme) was used for vector construction. All the primers used during these construction steps are listed in Table S1.

### Protoplast assay

The stem of *japonica* rice *cv* Zhonghua 11 was used to prepare the protoplasts in this study. Protoplasts were isolated from 12-day-old rice seedlings which were cultured at 28 °C on 1/2 MS medium with a 16-h light/8-h dark cycle. Rice transformation was performed as previously described (Ren et al. 2019). In each transfection, 20 μg plasmid DNA was introduced into approximately 1 × 10^6^/mL protoplasts by PEG-mediated transfection. The average transformation efficiency was 40–50%. The transfected protoplasts were incubated at 28 °C. 48 h after transfection, DNA from the protoplast was extracted using CTAB DNA extraction procedure. 1st round PCR amplification was performed using pfu polymerase (TransGen Biotech, Beijing, China) with 25 ng of genomic DNA as template. The PCR products amplified by the Hi-TOM primer sets (Table S1) were used for deep amplicon sequencing (Liu et al. 2019). Plasmids used for protoplasts transformation were extracted with EndoFree Maxi Plasmid Kit (Tiangen).

### *Agrobacterium*-mediated rice transformation

The constructed vectors were transformed into *Agrobacterium tumefaciens* strain EHA105 using the freeze–thaw method. Single colon of the *A. tumefaciens* strain was then cultivated in liquid LB medium (containing 100 mg/L kanamycin and 25 mg/L rifampicin) at 28 °C with shaking. After overnight incubation, 20 μL of fresh bacteria was transferred into 20 mL of fresh liquid LB medium (containing 100 mg/L kanamycin and 25 mg/L rifampicin), and fresh medium was incubated at 28 °C with shaking until the OD_600_ reached 0.6 ~ 1.0. The bacteria were harvested by centrifugation at 4200*g* for 10 min and then adjusted to an OD_600_ of 0.6 with transformation solution. Calli of a *japonica* rice (*cv* Zhonghua 11) grown in the culture were carefully placed in a sterile tube and were incubated with the *Agrobacterium* strains for 30 min. After incubation, the rice calli were transferred onto NB2C medium and co-cultivated for 3 days in dark at 28 °C.

After co-cultivation process, the calli were selected on 1st round selection medium containing 50 mg/L hygromycin and 300 mg/L timentin for two weeks at 28 °C in the dark. Then the well-grown calli were transferred to the 2nd round selection media containing 50 mg/L hygromycin and 300 mg/L timentin at 28 °C in dark for two weeks. After two rounds of selection, the vigorously resistant calli were transferred to regeneration media for about 3 ~ 4 weeks to regenerate green seedlings at 28 °C in the light (16 L: 8D). After regeneration, the green seedlings were transferred to the rooting medium to generate green plants at 28 °C in the light (16 L: 8D).

### Molecular characterization of the edited plants

Rice genomic DNA from approx. 0.2 g of leaf tissue was extracted using CTAB method. PCR amplification was performed using rTaq polymerase (Vazyme, Beijing, China) with 200 ng of genomic DNA as template. All plants were further genotyped by PCR and DNA sanger sequencing, and the sequencing results were analyzed using DsDecode (http://skl.scau.edu.cn/dsdecode/) (Liu et al. 2015). Some PCR products were also cloned into the blunt cloning vector P-Easy (Vazyme, Beijing, China), and at least 12 positive colonies for each sample were sequenced.

### RNA extraction and qPCR analysis

For rice plants, the total RNA was extraction using the RNeasy Plant Mini Kit (QIAGEN). DNase I (RNase-free) (New England Biolabs) was used to remove DNA from the total RNA samples. Approximately 1000–1500 ng of total RNA was used for complementary DNA synthesis with the SuperScript III First-Strand Synthesis Kit (Thermo Fisher). The AzuraQuant Green Fast qPCR Mix (Azura Genomics) coupled with the CFX96 Touch Real-Time PCR Detection System (Bio-Rad) was used to detect the transcript expression levels of target genes. *OsActin* was used as the endogenous control gene for rice. The fold changes of target genes were calculated by the 2^−ΔΔCt^ method. All primers used in this study are listed in Table S1.

### Supplementary Information

Below is the link to the electronic supplementary material.

Supplementary file 1 (DOC 5661 KB)

## Data Availability

All data generated in this study are available in the paper or online Supplementary Information.
