# Supplementary material for: Fusion of a rice endogenous N-methylpurine DNA glycosylase to a plant adenine base transition editor ABE8e enables A-to-K base editing in rice plants
Source: aBIOTECH. 2024 Mar 21;5(2):127–39. doi: 10.1007/s42994-024-00138-8 (PMC11224198; doi:10.1007/s42994-024-00138-8)
Supplement: Supplementary file 1 — (DOC 5661 KB) [file 42994_2024_138_MOESM1_ESM.doc]

**Supplemental Information**

**Fusion of a rice endogenous N-methylpurine DNA glycosylase to a plant adenine base transition editor ABE8e enabling A-to-K base editing in rice plants**

Yucai Li ^1, 2, 3, †^, Shaoya Li ^1, 3, †^, Chenfei Li ^1^, Chen Zhang ^1^, Lei Yan ^1^, Jingying Li ^1, 3^, Yubing He ^1, 3^, Yan Guo ^2^ and Lanqin Xia ^1, 3, *^

^1^ Institute of Crop Sciences (ICS), Chinese Academy of Agricultural Sciences (CAAS), Beijing 100081, China

^2^ State Key Laboratory of Plant Physiology and Biochemistry, College of Biological Sciences, China Agricultural University, Beijing 100193, China

^3^ Hainan Yazhou Bay Seed Laboratory/National Nanfan Research Institute (Sanya), CAAS, Sanya 572024, Hainan Province, China

**Contents**

**Fig. S1.** The genetic pedigree analyses of OsMPG: the phylogenetic analyses, predicted protein structure and amino acid sequence alignment of MPG

**Fig. S2.** The sequences of key elements used in rAKBEs

**Fig. S3.** The sequence chromatograms of different stable rice lines with A-to-K base transversions generated by rAKBE01 in T_0_ generation

**Fig. S4.** The sequence chromatograms of different stable rice lines with A-to-K base transversions generated by rAKBE04 in T_0_ generation

**Fig. S5.** The occurrence of small indels generated by rAKBE04 in rice stable lines in T_0_ generation.

**Fig. S6.** The relative expression levels of *OsDEP1* and *OsNRT1.1B* in edited lines and WT controls.

**Table S1.** The primer sets used in this study

**Table S2.** The rice endogenous targets used in this study

**Table S3.** The A-to-G base editing efficacies of different rAKBEs at different targeted loci in rice protoplast

**Table S4.** The A-to-Y base editing efficacies of different rAKBEs at different targeted loci in rice protoplast

**Table S5.** The frequencies of indels generated by different rAKBEs at different targeted loci in rice protoplast

**Table S6.** Analyses of potential off-target effects


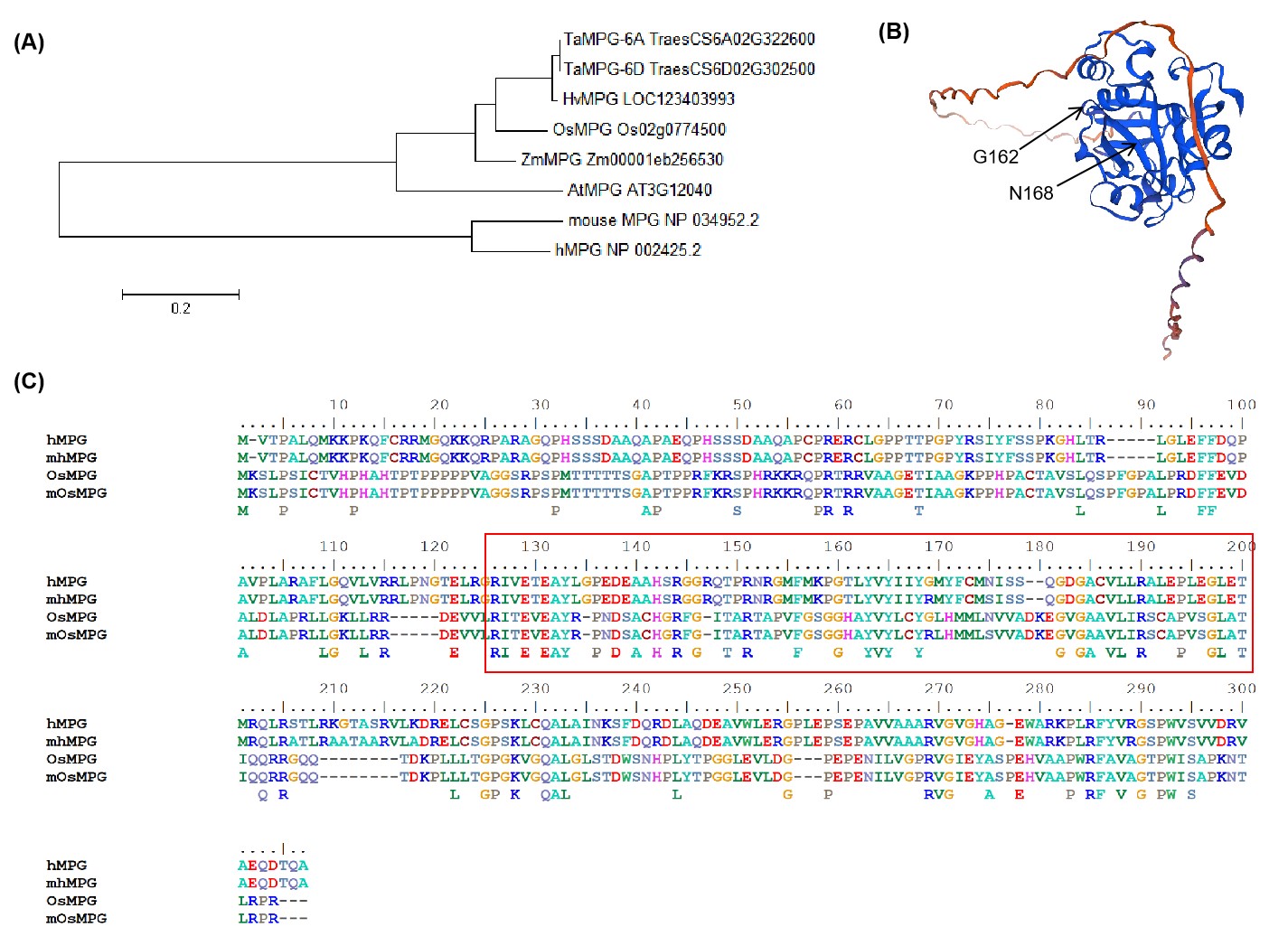


**Fig. S1. The genetic pedigree analyses of OsMPG: the phylogenetic analyses, predicted protein structure and amino acid sequence alignment of MPG.**

(A) The phylogenetic analyses of MPG in rice and other plant species. OsMPG, MPG from rice; TaMPG-6A, MPG from wheat; TaMPG-6D, MPG from wheat; HvMPG, MPG from barley; ZmMPG, MPG from maize; AtMPG, MPG from Arabidopsis thaliana; mouse MPG, MPG from jimpy mice; hMPG，MPG from human. (B) A predicted protein structure of OsMPG. The protein structure is constructed using the PROTEIN DATA BANK server (http://www.rcsb.org), the mutated residues contributing to the improved efficiency of A-to-Y base editing are labeled with arrow. (C) Amino acid sequence alignments of hMPG, mhMPG, OsMPG and mOsMPG. The different amino acids were used distinct background colors and the conserved positions were indicated by boxes.

**Fig. S2. The sequences of key elements used in rAKBEs**

| >**rAKBE01, NLS-TadA8e-linker(32aa)-nCas9(D10A)-linker(13aa)-OsMPG-NLS**  ATGCCGAAGAAGAAGAGGAAGGTTGGCATCCACGGGGTGCCAGCTGCTTCAGAAGTCGAGTTCTCCCATGAGTATTGGATGAGGCACGCCCTCACTCTTGCGAAGAGGGCCAGGGACGAGAGGGAGGTGCCGGTCGGTGCTGTCCTGGTCTTGAATAACAGGGTGATAGGCGAAGGTTGGAACAGGGCTATTGGCCTTCATGACCCTACTGCTCATGCGGAAATCATGGCACTTAGACAGGGGGGCCTCGTTATGCAAAATTACCGCCTGATCGACGCCACTCTTTATGTCACATTTGAACCATGTGTTATGTGTGCGGGCGCTATGATCCATTCACGCATAGGTCGCGTGGTTTTTGGAGTTCGCAACTCGAAAAGAGGGGCTGCAGGCTCTCTGATGAACGTTTTGAACTATCCGGGAATGAACCATAGAGTCGAAATCACAGAAGGGATTTTGGCAGACGAATGCGCGGCTCTTCTTTGTGACTTTTACAGAATGCCCCGCCAAGTGTTTAATGCTCAAAAGAAAGCGCAGAGTAGCATCAACTCCGGCGGCTCATCTGGCGGCTCCAGCGGTTCAGAGACACCTGGCACATCAGAGTCTGCCACACCAGAGTCATCTGGCGGCTCCAGCGGCGGCAGCGACAAGAAGTACTCGATCGGCCTCGCCATTGGGACTAACTCTGTTGGCTGGGCCGTGATCACCGACGAGTACAAGGTGCCCTCAAAGAAGTTCAAGGTCCTGGGCAACACCGATCGGCATTCCATCAAGAAGAATCTCATTGGCGCTCTCCTGTTCGACAGCGGCGAGACGGCTGAGGCTACGCGGCTCAAGCGCACCGCCCGCAGGCGGTACACGCGCAGGAAGAATCGCATCTGCTACCTGCAGGAGATTTTCTCCAACGAGATGGCGAAGGTTGACGATTCTTTCTTCCACAGGCTGGAGGAGTCATTCCTCGTGGAGGAGGATAAGAAGCACGAGCGGCATCCAATCTTCGGCAACATTGTCGACGAGGTTGCCTACCACGAGAAGTACCCTACGATCTACCATCTGCGGAAGAAGCTCGTGGACTCCACAGATAAGGCGGACCTCCGCCTGATCTACCTCGCTCTGGCCCACATGATTAAGTTCAGGGGCCATTTCCTGATCGAGGGGGATCTCAACCCGGACAATAGCGATGTTGACAAGCTGTTCATCCAGCTCGTGCAGACGTACAACCAGCTCTTCGAGGAGAACCCCATTAATGCGTCAGGCGTCGACGCGAAGGCTATCCTGTCCGCTAGGCTCTCGAAGTCTCGGCGCCTCGAGAACCTGATCGCCCAGCTGCCGGGCGAGAAGAAGAACGGCCTGTTCGGGAATCTCATTGCGCTCAGCCTGGGGCTCACGCCCAACTTCAAGTCGAATTTCGATCTCGCTGAGGACGCCAAGCTGCAGCTCTCCAAGGACACATACGACGATGACCTGGATAACCTCCTGGCCCAGATCGGCGATCAGTACGCGGACCTGTTCCTCGCTGCCAAGAATCTGTCGGACGCCATCCTCCTGTCTGATATTCTCAGGGTGAACACCGAGATTACGAAGGCTCCGCTCTCAGCCTCCATGATCAAGCGCTACGACGAGCACCATCAGGATCTGACCCTCCTGAAGGCGCTGGTCAGGCAGCAGCTCCCCGAGAAGTACAAGGAGATCTTCTTCGATCAGTCGAAGAACGGCTACGCTGGGTACATTGACGGCGGGGCCTCTCAGGAGGAGTTCTACAAGTTCATCAAGCCGATTCTGGAGAAGATGGACGGCACGGAGGAGCTGCTGGTGAAGCTCAATCGCGAGGACCTCCTGAGGAAGCAGCGGACATTCGATAACGGCAGCATCCCACACCAGATTCATCTCGGGGAGCTGCACGCTATCCTGAGGAGGCAGGAGGACTTCTACCCTTTCCTCAAGGATAACCGCGAGAAGATCGAGAAGATTCTGACTTTCAGGATCCCGTACTACGTCGGCCCACTCGCTAGGGGCAACTCCCGCTTCGCTTGGATGACCCGCAAGTCAGAGGAGACGATCACGCCGTGGAACTTCGAGGAGGTGGTCGACAAGGGCGCTAGCGCTCAGTCGTTCATCGAGAGGATGACGAATTTCGACAAGAACCTGCCAAATGAGAAGGTGCTCCCTAAGCACTCGCTCCTGTACGAGTACTTCACAGTCTACAACGAGCTGACTAAGGTGAAGTATGTGACCGAGGGCATGAGGAAGCCGGCTTTCCTGTCTGGGGAGCAGAAGAAGGCCATCGTGGACCTCCTGTTCAAGACCAACCGGAAGGTCACGGTTAAGCAGCTCAAGGAGGACTACTTCAAGAAGATTGAGTGCTTCGATTCGGTCGAGATCTCTGGCGTTGAGGACCGCTTCAACGCCTCCCTGGGGACCTACCACGATCTCCTGAAGATCATTAAGGATAAGGACTTCCTGGACAACGAGGAGAATGAGGATATCCTCGAGGACATTGTGCTGACACTCACTCTGTTCGAGGACCGGGAGATGATCGAGGAGCGCCTGAAGACTTACGCCCATCTCTTCGATGACAAGGTCATGAAGCAGCTCAAGAGGAGGAGGTACACCGGCTGGGGGAGGCTGAGCAGGAAGCTCATCAACGGCATTCGGGACAAGCAGTCCGGGAAGACGATCCTCGACTTCCTGAAGAGCGATGGCTTCGCGAACCGCAATTTCATGCAGCTGATTCACGATGACAGCCTCACATTCAAGGAGGATATCCAGAAGGCTCAGGTGAGCGGCCAGGGGGACTCGCTGCACGAGCATATCGCGAACCTCGCTGGCTCGCCAGCTATCAAGAAGGGGATTCTGCAGACCGTGAAGGTTGTGGACGAGCTGGTGAAGGTCATGGGCAGGCACAAGCCTGAGAACATCGTCATTGAGATGGCCCGGGAGAATCAGACCACGCAGAAGGGCCAGAAGAACTCACGCGAGAGGATGAAGAGGATCGAGGAGGGCATTAAGGAGCTGGGGTCCCAGATCCTCAAGGAGCACCCGGTGGAGAACACGCAGCTGCAGAATGAGAAGCTCTACCTGTACTACCTCCAGAATGGCCGCGATATGTATGTGGACCAGGAGCTGGATATTAACAGGCTCAGCGATTACGACGTCGATCATATCGTTCCACAGTCATTCCTGAAGGATGACTCCATTGACAACAAGGTCCTCACCAGGTCGGACAAGAACCGGGGCAAGTCTGATAATGTTCCTTCAGAGGAGGTCGTTAAGAAGATGAAGAACTACTGGCGCCAGCTCCTGAATGCCAAGCTGATCACGCAGCGGAAGTTCGATAACCTCACAAAGGCTGAGAGGGGCGGGCTCTCTGAGCTGGACAAGGCGGGCTTCATCAAGAGGCAGCTGGTCGAGACACGGCAGATCACTAAGCACGTTGCGCAGATTCTCGACTCACGGATGAACACTAAGTACGATGAGAATGACAAGCTGATCCGCGAGGTGAAGGTCATCACCCTGAAGTCAAAGCTCGTCTCCGACTTCAGGAAGGATTTCCAGTTCTACAAGGTTCGGGAGATCAACAATTACCACCATGCCCATGACGCGTACCTGAACGCGGTGGTCGGCACAGCTCTGATCAAGAAGTACCCAAAGCTCGAGAGCGAGTTCGTGTACGGGGACTACAAGGTTTACGATGTGAGGAAGATGATCGCCAAGTCGGAGCAGGAGATTGGCAAGGCTACCGCCAAGTACTTCTTCTACTCTAACATTATGAATTTCTTCAAGACAGAGATCACTCTGGCCAATGGCGAGATCCGGAAGCGCCCCCTCATCGAGACGAACGGCGAGACGGGGGAGATCGTGTGGGACAAGGGCAGGGATTTCGCGACCGTCAGGAAGGTTCTCTCCATGCCACAAGTGAATATCGTCAAGAAGACAGAGGTCCAGACTGGCGGGTTCTCTAAGGAGTCAATTCTGCCTAAGCGGAACAGCGACAAGCTCATCGCCCGCAAGAAGGACTGGGATCCGAAGAAGTACGGCGGGTTCGACAGCCCCACTGTGGCCTACTCGGTCCTGGTTGTGGCGAAGGTTGAGAAGGGCAAGTCCAAGAAGCTCAAGAGCGTGAAGGAGCTGCTGGGGATCACGATTATGGAGCGCTCCAGCTTCGAGAAGAACCCGATCGATTTCCTGGAGGCGAAGGGCTACAAGGAGGTGAAGAAGGACCTGATCATTAAGCTCCCCAAGTACTCACTCTTCGAGCTGGAGAACGGCAGGAAGCGGATGCTGGCTTCCGCTGGCGAGCTGCAGAAGGGGAACGAGCTGGCTCTGCCGTCCAAGTATGTGAACTTCCTCTACCTGGCCTCCCACTACGAGAAGCTCAAGGGCAGCCCCGAGGACAACGAGCAGAAGCAGCTGTTCGTCGAGCAGCACAAGCATTACCTCGACGAGATCATTGAGCAGATTTCCGAGTTCTCCAAGCGCGTGATCCTGGCCGACGCGAATCTGGATAAGGTCCTCTCCGCGTACAACAAGCACCGCGACAAGCCAATCAGGGAGCAGGCTGAGAATATCATTCATCTCTTCACCCTGACGAACCTCGGCGCCCCTGCTGCTTTCAAGTACTTCGACACAACTATCGATCGCAAGAGGTACACAAGCACTAAGGAGGTCCTGGACGCGACCCTCATCCACCAGTCGATTACCGGCCTCTACGAGACGCGCATCGACCTGTCTCAGCTCGGGGGCGACTCCGGCGGCTCTGGCGGCTCCGGCGGCTCCGGGGGGAGCAAATCCCTTCCTTCCATCTGCACCGTCCATCCCCACGCACACACCCCCACGCCACCGCCGCCGCCGGTCGCCGGAGGGAGCCGGCCATCTCCGATGACCACCACCACCACCTCCGGGGCGCCCACGCCCCCACGCTTCAAGCGATCCCCCCACAGGAAGAAGCGGCAGCCCCGTACCCGCCGCGTCGCCGCCGGAGAGACCATCGCCGCGGGGAAACCACCGCACCCGGCGTGTACGGCGGTGTCGTTGCAGTCGCCGTTTGGGCCCGCATTGCCGCGCGATTTCTTCGAGGTGGACGCGCTTGACCTCGCCCCACGCCTCCTCGGCAAGCTGCTGCGCCGCGACGAAGTCGTCCTCCGCATCACCGAGGTGGAGGCTTACAGGCCAAATGACTCCGCGTGCCACGGCCGGTTCGGCATCACGGCGAGGACTGCTCCTGTGTTTGGATCAGGAGGGCATGCGTATGTTTACCTGTGCTATGGACTGCACATGATGCTCAATGTCGTCGCTGACAAGGAGGGAGTTGGAGCTGCTGTTCTGATTCGGTCATGTGCTCCCGTTAGTGGGCTGGCAACTATTCAGCAGCGTCGAGGCCAGCAGACTGATAAGCCACTTCTACTCACTGGACCAGGAAAGGTTGGTCAAGCTCTGGGGCTTTCCACTGACTGGTCCAACCATCCTCTGTACACACCTGGTGGGTTGGAGGTACTAGACGGGCCAGAACCGGAGAACATTTTGGTTGGCCCCCGTGTAGGCATCGAATACGCATCGCCGGAGCATGTTGCTGCACCATGGAGGTTCGCCGTCGCAGGGACGCCATGGATTAGTGCCCCCAAGAACACTCTCAGACCAAGGAAGAGGCCGGCGGCGACCAAGAAGGCGGGCCAAGCTAAGAAGAAGAAGTGA |
| --- |
| > **rAKBE03, NLS-VP64- linker(32aa)-TadA8e-linker(32aa)-nCas9(D10A)-linker(13aa)-OsMPG-NLS**  ATGCCGAAGAAGAAGAGGAAGGTTGGCATCCACGGGGTGCCAGCTGCTGACGCCCTCGACGACTTCGATCTCGACATGCTCGGCTCCGACGCCCTCGATGATTTCGACCTCGACATGCTGGGCTCCGACGCTCTCGATGATTTCGATCTCGATATGCTCGGCTCTGATGCCCTCGACGATTTCGACCTGGACATGCTCTCCGGCGGCTCTTCTGGCGGCTCTTCCGGCTCTGAGACTCCAGGCACTTCCGAGTCTGCCACCCCTGAGTCTTCCGGCGGCTCCTCTGGCGGCTCCTCAGAAGTCGAGTTCTCCCATGAGTATTGGATGAGGCACGCCCTCACTCTTGCGAAGAGGGCCAGGGACGAGAGGGAGGTGCCGGTCGGTGCTGTCCTGGTCTTGAATAACAGGGTGATAGGCGAAGGTTGGAACAGGGCTATTGGCCTTCATGACCCTACTGCTCATGCGGAAATCATGGCACTTAGACAGGGGGGCCTCGTTATGCAAAATTACCGCCTGATCGACGCCACTCTTTATGTCACATTTGAACCATGTGTTATGTGTGCGGGCGCTATGATCCATTCACGCATAGGTCGCGTGGTTTTTGGAGTTCGCAACTCGAAAAGAGGGGCTGCAGGCTCTCTGATGAACGTTTTGAACTATCCGGGAATGAACCATAGAGTCGAAATCACAGAAGGGATTTTGGCAGACGAATGCGCGGCTCTTCTTTGTGACTTTTACAGAATGCCCCGCCAAGTGTTTAATGCTCAAAAGAAAGCGCAGAGTAGCATCAACTCCGGCGGCTCATCTGGCGGCTCCAGCGGTTCAGAGACACCTGGCACATCAGAGTCTGCCACACCAGAGTCATCTGGCGGCTCCAGCGGCGGCAGCGACAAGAAGTACTCGATCGGCCTCGCCATTGGGACTAACTCTGTTGGCTGGGCCGTGATCACCGACGAGTACAAGGTGCCCTCAAAGAAGTTCAAGGTCCTGGGCAACACCGATCGGCATTCCATCAAGAAGAATCTCATTGGCGCTCTCCTGTTCGACAGCGGCGAGACGGCTGAGGCTACGCGGCTCAAGCGCACCGCCCGCAGGCGGTACACGCGCAGGAAGAATCGCATCTGCTACCTGCAGGAGATTTTCTCCAACGAGATGGCGAAGGTTGACGATTCTTTCTTCCACAGGCTGGAGGAGTCATTCCTCGTGGAGGAGGATAAGAAGCACGAGCGGCATCCAATCTTCGGCAACATTGTCGACGAGGTTGCCTACCACGAGAAGTACCCTACGATCTACCATCTGCGGAAGAAGCTCGTGGACTCCACAGATAAGGCGGACCTCCGCCTGATCTACCTCGCTCTGGCCCACATGATTAAGTTCAGGGGCCATTTCCTGATCGAGGGGGATCTCAACCCGGACAATAGCGATGTTGACAAGCTGTTCATCCAGCTCGTGCAGACGTACAACCAGCTCTTCGAGGAGAACCCCATTAATGCGTCAGGCGTCGACGCGAAGGCTATCCTGTCCGCTAGGCTCTCGAAGTCTCGGCGCCTCGAGAACCTGATCGCCCAGCTGCCGGGCGAGAAGAAGAACGGCCTGTTCGGGAATCTCATTGCGCTCAGCCTGGGGCTCACGCCCAACTTCAAGTCGAATTTCGATCTCGCTGAGGACGCCAAGCTGCAGCTCTCCAAGGACACATACGACGATGACCTGGATAACCTCCTGGCCCAGATCGGCGATCAGTACGCGGACCTGTTCCTCGCTGCCAAGAATCTGTCGGACGCCATCCTCCTGTCTGATATTCTCAGGGTGAACACCGAGATTACGAAGGCTCCGCTCTCAGCCTCCATGATCAAGCGCTACGACGAGCACCATCAGGATCTGACCCTCCTGAAGGCGCTGGTCAGGCAGCAGCTCCCCGAGAAGTACAAGGAGATCTTCTTCGATCAGTCGAAGAACGGCTACGCTGGGTACATTGACGGCGGGGCCTCTCAGGAGGAGTTCTACAAGTTCATCAAGCCGATTCTGGAGAAGATGGACGGCACGGAGGAGCTGCTGGTGAAGCTCAATCGCGAGGACCTCCTGAGGAAGCAGCGGACATTCGATAACGGCAGCATCCCACACCAGATTCATCTCGGGGAGCTGCACGCTATCCTGAGGAGGCAGGAGGACTTCTACCCTTTCCTCAAGGATAACCGCGAGAAGATCGAGAAGATTCTGACTTTCAGGATCCCGTACTACGTCGGCCCACTCGCTAGGGGCAACTCCCGCTTCGCTTGGATGACCCGCAAGTCAGAGGAGACGATCACGCCGTGGAACTTCGAGGAGGTGGTCGACAAGGGCGCTAGCGCTCAGTCGTTCATCGAGAGGATGACGAATTTCGACAAGAACCTGCCAAATGAGAAGGTGCTCCCTAAGCACTCGCTCCTGTACGAGTACTTCACAGTCTACAACGAGCTGACTAAGGTGAAGTATGTGACCGAGGGCATGAGGAAGCCGGCTTTCCTGTCTGGGGAGCAGAAGAAGGCCATCGTGGACCTCCTGTTCAAGACCAACCGGAAGGTCACGGTTAAGCAGCTCAAGGAGGACTACTTCAAGAAGATTGAGTGCTTCGATTCGGTCGAGATCTCTGGCGTTGAGGACCGCTTCAACGCCTCCCTGGGGACCTACCACGATCTCCTGAAGATCATTAAGGATAAGGACTTCCTGGACAACGAGGAGAATGAGGATATCCTCGAGGACATTGTGCTGACACTCACTCTGTTCGAGGACCGGGAGATGATCGAGGAGCGCCTGAAGACTTACGCCCATCTCTTCGATGACAAGGTCATGAAGCAGCTCAAGAGGAGGAGGTACACCGGCTGGGGGAGGCTGAGCAGGAAGCTCATCAACGGCATTCGGGACAAGCAGTCCGGGAAGACGATCCTCGACTTCCTGAAGAGCGATGGCTTCGCGAACCGCAATTTCATGCAGCTGATTCACGATGACAGCCTCACATTCAAGGAGGATATCCAGAAGGCTCAGGTGAGCGGCCAGGGGGACTCGCTGCACGAGCATATCGCGAACCTCGCTGGCTCGCCAGCTATCAAGAAGGGGATTCTGCAGACCGTGAAGGTTGTGGACGAGCTGGTGAAGGTCATGGGCAGGCACAAGCCTGAGAACATCGTCATTGAGATGGCCCGGGAGAATCAGACCACGCAGAAGGGCCAGAAGAACTCACGCGAGAGGATGAAGAGGATCGAGGAGGGCATTAAGGAGCTGGGGTCCCAGATCCTCAAGGAGCACCCGGTGGAGAACACGCAGCTGCAGAATGAGAAGCTCTACCTGTACTACCTCCAGAATGGCCGCGATATGTATGTGGACCAGGAGCTGGATATTAACAGGCTCAGCGATTACGACGTCGATCATATCGTTCCACAGTCATTCCTGAAGGATGACTCCATTGACAACAAGGTCCTCACCAGGTCGGACAAGAACCGGGGCAAGTCTGATAATGTTCCTTCAGAGGAGGTCGTTAAGAAGATGAAGAACTACTGGCGCCAGCTCCTGAATGCCAAGCTGATCACGCAGCGGAAGTTCGATAACCTCACAAAGGCTGAGAGGGGCGGGCTCTCTGAGCTGGACAAGGCGGGCTTCATCAAGAGGCAGCTGGTCGAGACACGGCAGATCACTAAGCACGTTGCGCAGATTCTCGACTCACGGATGAACACTAAGTACGATGAGAATGACAAGCTGATCCGCGAGGTGAAGGTCATCACCCTGAAGTCAAAGCTCGTCTCCGACTTCAGGAAGGATTTCCAGTTCTACAAGGTTCGGGAGATCAACAATTACCACCATGCCCATGACGCGTACCTGAACGCGGTGGTCGGCACAGCTCTGATCAAGAAGTACCCAAAGCTCGAGAGCGAGTTCGTGTACGGGGACTACAAGGTTTACGATGTGAGGAAGATGATCGCCAAGTCGGAGCAGGAGATTGGCAAGGCTACCGCCAAGTACTTCTTCTACTCTAACATTATGAATTTCTTCAAGACAGAGATCACTCTGGCCAATGGCGAGATCCGGAAGCGCCCCCTCATCGAGACGAACGGCGAGACGGGGGAGATCGTGTGGGACAAGGGCAGGGATTTCGCGACCGTCAGGAAGGTTCTCTCCATGCCACAAGTGAATATCGTCAAGAAGACAGAGGTCCAGACTGGCGGGTTCTCTAAGGAGTCAATTCTGCCTAAGCGGAACAGCGACAAGCTCATCGCCCGCAAGAAGGACTGGGATCCGAAGAAGTACGGCGGGTTCGACAGCCCCACTGTGGCCTACTCGGTCCTGGTTGTGGCGAAGGTTGAGAAGGGCAAGTCCAAGAAGCTCAAGAGCGTGAAGGAGCTGCTGGGGATCACGATTATGGAGCGCTCCAGCTTCGAGAAGAACCCGATCGATTTCCTGGAGGCGAAGGGCTACAAGGAGGTGAAGAAGGACCTGATCATTAAGCTCCCCAAGTACTCACTCTTCGAGCTGGAGAACGGCAGGAAGCGGATGCTGGCTTCCGCTGGCGAGCTGCAGAAGGGGAACGAGCTGGCTCTGCCGTCCAAGTATGTGAACTTCCTCTACCTGGCCTCCCACTACGAGAAGCTCAAGGGCAGCCCCGAGGACAACGAGCAGAAGCAGCTGTTCGTCGAGCAGCACAAGCATTACCTCGACGAGATCATTGAGCAGATTTCCGAGTTCTCCAAGCGCGTGATCCTGGCCGACGCGAATCTGGATAAGGTCCTCTCCGCGTACAACAAGCACCGCGACAAGCCAATCAGGGAGCAGGCTGAGAATATCATTCATCTCTTCACCCTGACGAACCTCGGCGCCCCTGCTGCTTTCAAGTACTTCGACACAACTATCGATCGCAAGAGGTACACAAGCACTAAGGAGGTCCTGGACGCGACCCTCATCCACCAGTCGATTACCGGCCTCTACGAGACGCGCATCGACCTGTCTCAGCTCGGGGGCGACTCCGGCGGCTCTGGCGGCTCCGGCGGCTCCGGGGGGAGCAAATCCCTTCCTTCCATCTGCACCGTCCATCCCCACGCACACACCCCCACGCCACCGCCGCCGCCGGTCGCCGGAGGGAGCCGGCCATCTCCGATGACCACCACCACCACCTCCGGGGCGCCCACGCCCCCACGCTTCAAGCGATCCCCCCACAGGAAGAAGCGGCAGCCCCGTACCCGCCGCGTCGCCGCCGGAGAGACCATCGCCGCGGGGAAACCACCGCACCCGGCGTGTACGGCGGTGTCGTTGCAGTCGCCGTTTGGGCCCGCATTGCCGCGCGATTTCTTCGAGGTGGACGCGCTTGACCTCGCCCCACGCCTCCTCGGCAAGCTGCTGCGCCGCGACGAAGTCGTCCTCCGCATCACCGAGGTGGAGGCTTACAGGCCAAATGACTCCGCGTGCCACGGCCGGTTCGGCATCACGGCGAGGACTGCTCCTGTGTTTGGATCAGGAGGGCATGCGTATGTTTACCTGTGCTATGGACTGCACATGATGCTCAATGTCGTCGCTGACAAGGAGGGAGTTGGAGCTGCTGTTCTGATTCGGTCATGTGCTCCCGTTAGTGGGCTGGCAACTATTCAGCAGCGTCGAGGCCAGCAGACTGATAAGCCACTTCTACTCACTGGACCAGGAAAGGTTGGTCAAGCTCTGGGGCTTTCCACTGACTGGTCCAACCATCCTCTGTACACACCTGGTGGGTTGGAGGTACTAGACGGGCCAGAACCGGAGAACATTTTGGTTGGCCCCCGTGTAGGCATCGAATACGCATCGCCGGAGCATGTTGCTGCACCATGGAGGTTCGCCGTCGCAGGGACGCCATGGATTAGTGCCCCCAAGAACACTCTCAGACCAAGGAAGAGGCCGGCGGCGACCAAGAAGGCGGGCCAAGCTAAGAAGAAGAAGTGA |
| >**mOsMPG, the bases shadowed in red represent the mutation sites**  ATGAAATCCCTTCCTTCCATCTGCACCGTCCATCCCCACGCACACACCCCCACGCCACCGCCGCCGCCGGTCGCCGGAGGGAGCCGGCCATCTCCGATGACCACCACCACCACCTCCGGGGCGCCCACGCCCCCACGCTTCAAGCGATCCCCCCACAGGAAGAAGCGGCAGCCCCGTACCCGCCGCGTCGCCGCCGGAGAGACCATCGCCGCGGGGAAACCACCGCACCCGGCGTGTACGGCGGTGTCGTTGCAGTCGCCGTTTGGGCCCGCATTGCCGCGCGATTTCTTCGAGGTGGACGCGCTTGACCTCGCCCCACGCCTCCTCGGCAAGCTGCTGCGCCGCGACGAAGTCGTCCTCCGCATCACCGAGGTGGAGGCTTACAGGCCAAATGACTCCGCGTGCCACGGCCGGTTCGGCATCACGGCGAGGACTGCTCCTGTGTTTGGATCAGGAGGGCATGCGTATGTTTACCTGTGCTATCGCCTGCACATGATGCTCTCCGTCGTCGCTGACAAGGAGGGAGTTGGAGCTGCTGTTCTGATTCGGTCATGTGCTCCCGTTAGTGGGCTGGCAACTATTCAGCAGCGTCGAGGCCAGCAGACTGATAAGCCACTTCTACTCACTGGACCAGGAAAGGTTGGTCAAGCTCTGGGGCTTTCCACTGACTGGTCCAACCATCCTCTGTACACACCTGGTGGGTTGGAGGTACTAGACGGGCCAGAACCGGAGAACATTTTGGTTGGCCCCCGTGTAGGCATCGAATACGCATCGCCGGAGCATGTTGCTGCACCATGGAGGTTCGCCGTCGCAGGGACGCCATGGATTAGTGCCCCCAAGAACACTCTCAGACCAAGG |

**
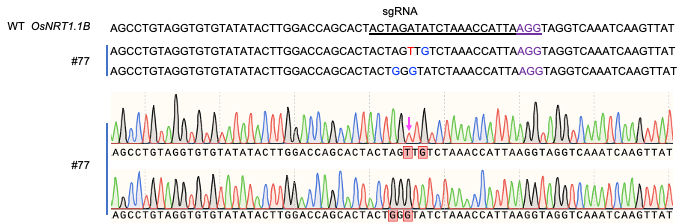
**

**
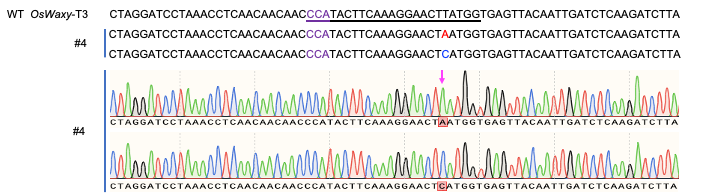
**

**Fig. S3.** **The sequence chromatograms of different stable rice lines with A-to-Y base transversions generated by rAKBE01 in T_0_ generation**

A-to-T transversion was shown in red, and A-to-G transitions was shown in blue. The sgRNA target sequences are underlined and their PAM sites are in purple.

**
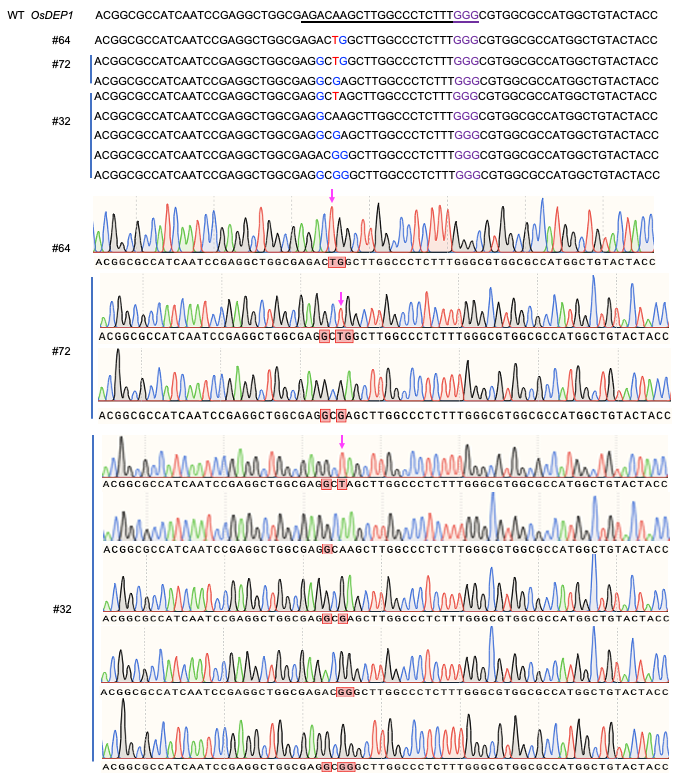
**

**
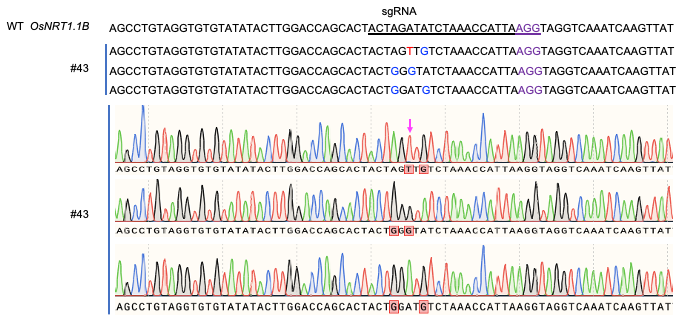
**

**
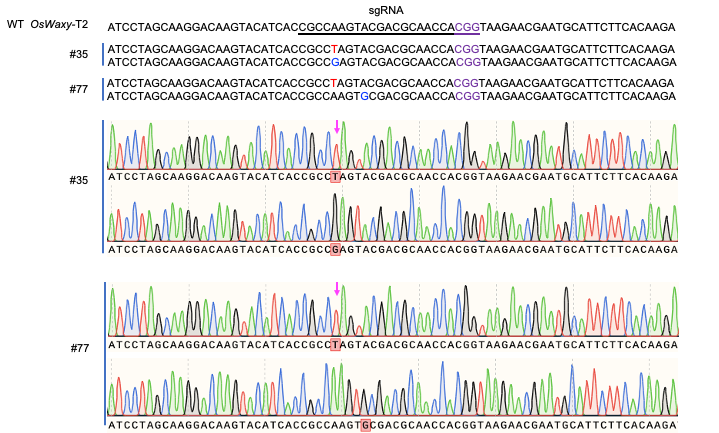
**

**
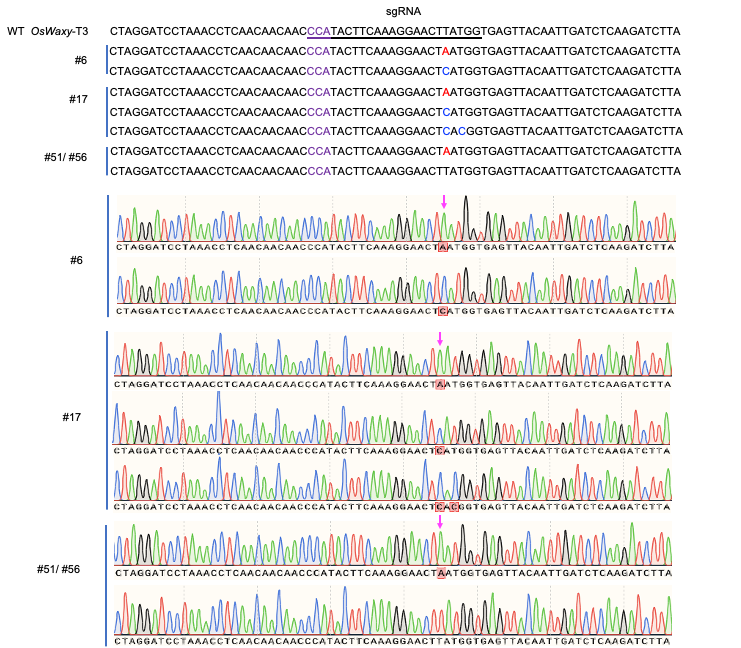
**

**
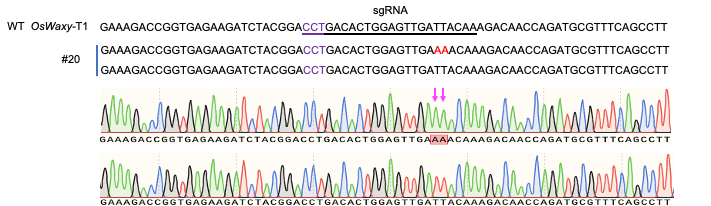
**

**Fig. S4.** **The sequence chromatograms of different stable rice lines with A-to-Y base transversions generated by rAKBE04 in T_0_ generation**

A-to-T transversion was shown in red, and A-to-G transitions was shown in blue. The sgRNA target sequences are underlined and their PAM sites are in purple.

**
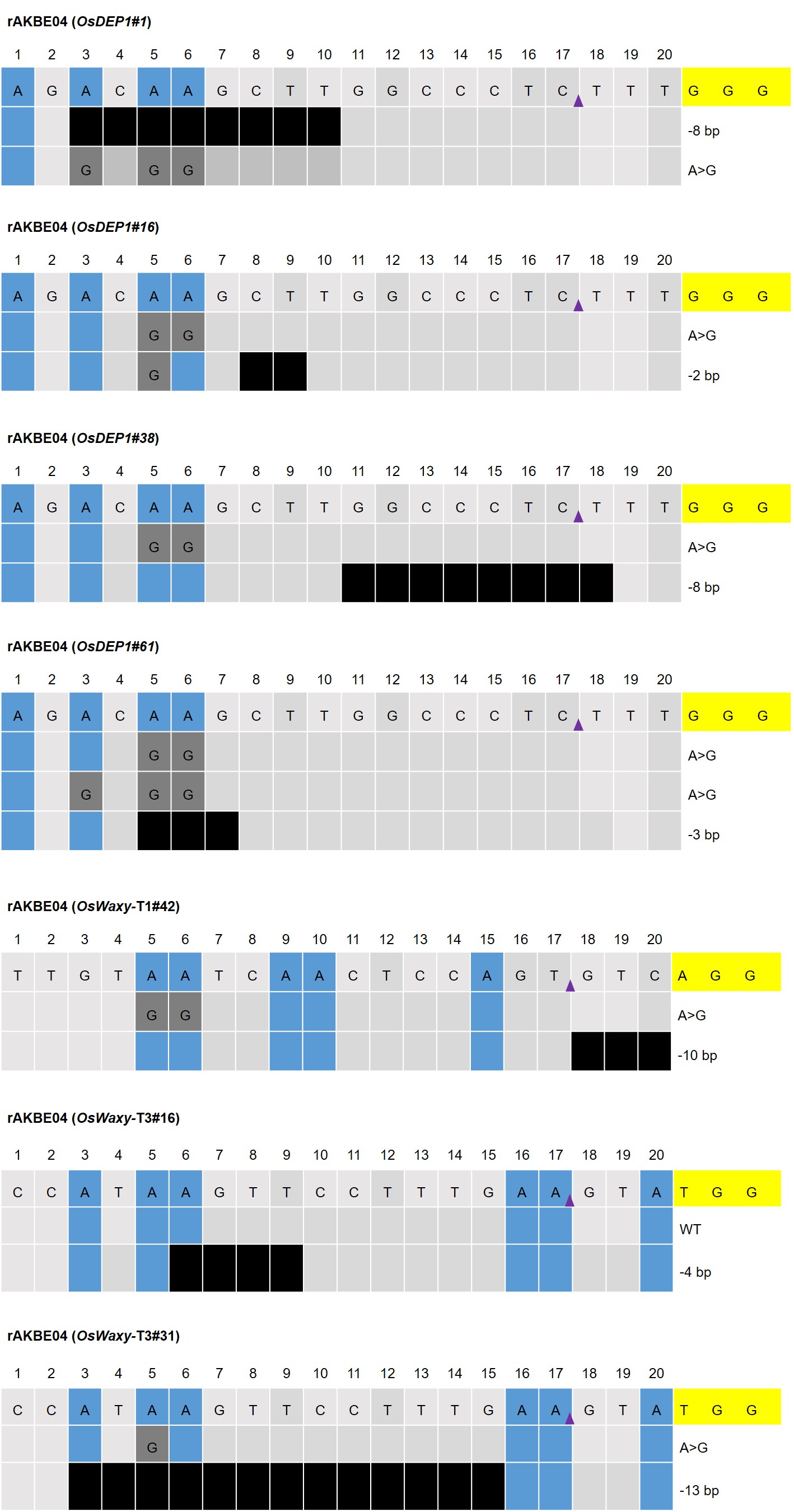
**

**
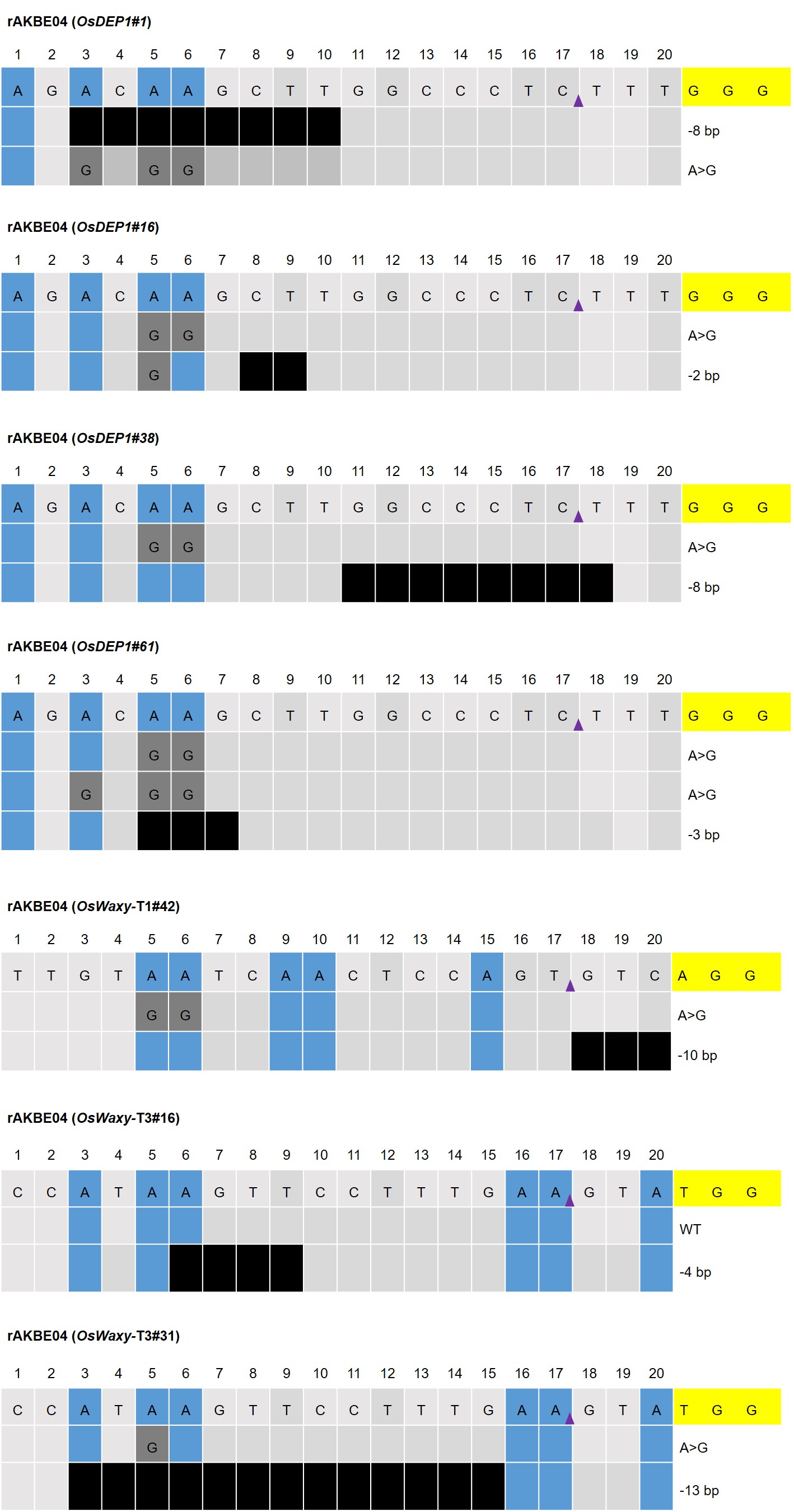
**

**Fig. S5. The occurrence of small indels generated by rAKBE04 in rice stable lines in T_0_ generation**

The indels efficiency and uniform precise deletion ratio are shown below PAM. PAMs are highlighted in yellow, and black bars represent the deleted nucleotide fragment. Red bars represent the inserted nucleotides. “-”, deletion; “+”, insertion.

**
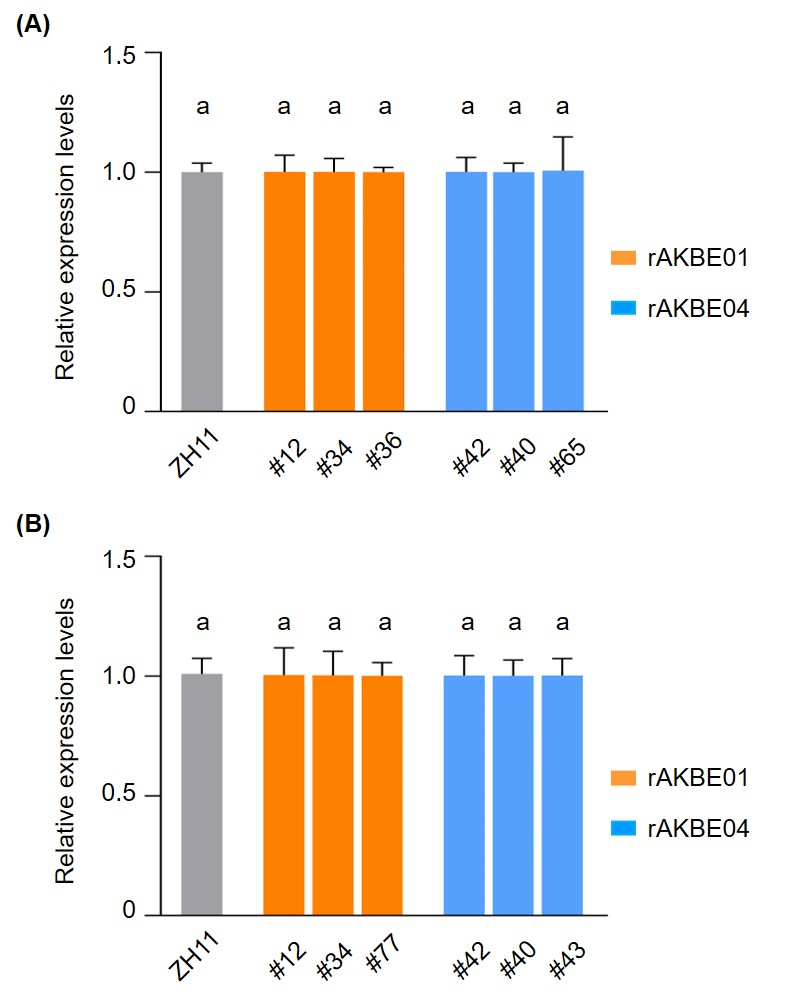
**

**Fig. S6. The relative expression levels of edited lines and WT controls were measured by quantitative PCR with reverse transcription (RT–qPCR) with three biological and three technical replicates per biological sample.**

(A) The relative expression levels of *OsDEP1* edited lines and WT controls. (B) The relative expression levels of *OsNRT1.1B* edited lines and WT controls.

**Table S1. The primer sets used in this study**

| **Primer Name** | **Primer Sequence（5′ to 3′）** | **Application** | **Annealing (℃)** | **Fragment size (bp)** |
| --- | --- | --- | --- | --- |
| OsMPG-F | CATCGATGAAATCCCTTCCTTCC | Amplification of OsMPG | 58 | 868 |
| OsMPG-R | CCCTCACCTTGGTCTGAGAG |  |  |  |
| *Avr*II-F | GGTGTTACTTCTGCAGGCCACCCCCTAGGCCTACATGGCTCCGAAGAAGA | Construction of ABE8e plasmid | 58 | 948 |
| *Sbf* I-R | GCCATCTCGTTGGAGAAAATCTCCTGCAGG |  |  |  |
| *Sac* I-OsMPG-F | CTGTCTCAGCTCGGGGGCGACTCCGGCGGCTCTGGCGGCTCCGGCGGCTCCGGGGGGAGCAAATCCCTTCCTTCCATCTGC | Construction of rAKBE01 plasmid | 58 | 991 |
| *Sac* I-OsMPG-R | GATGATACGAACGAAAGCTCTGAGCTCACTTCTTCTTCTTAGCTTGGCCC |  |  |  |
| *Sac* I-OsMPG-F | CTGTCTCAGCTCGGGGGCGACTCCGGCGGCTCTGGCGGCTCCGGCGGCTCCGGGGGGAGCAAATCCCTTCCTTCCATCTGC | Construction of rAKBE02 plasmid | 58 | 574 |
| OsMPG 162-R | GCGACGACGGAGAGCATCATGTGCAGGCGATAGCACAGGTAAACATACGC |  |  |  |
| OsMPG 168-F | CCTGCACATGATGCTCTCCGTCGTCGCTGACAAGGAGGGAGTTGGAGCTG | Construction of rAKBE02 plasmid | 58 | 449 |
| *Sac*I-OsMPG-R | GATGATACGAACGAAAGCTCTGAGCTCACTTCTTCTTCTTAGCTTGGCCC |  |  |  |
| VP64-F | CAGGCCACCCCCTAGGCCTACATGGCTCCAAAGAAGAAGCGCAAGGTGGACGCCCTCGA | Construction of rAKBE03 plasmid | 58 | 315 |
| VP64-R | ATGGGAGAACTCGACTTCTGAGGAGCCGCCAGAGGAGC |  |  |  |
| TadA 8e-F | TCCTCTGGCGGCTCCTCAGAAGTCGAGTTCTC |  | 58 | 878 |
| *Sbf* I-R | GCCATCTCGTTGGAGAAAATCTCCTGCAGG |  |  |  |
| VP64-F | CAGGCCACCCCCTAGGCCTACATGGCTCCAAAGAAGAAGCGCAAGGTGGACGCCCTCGA | Construction of rAKBE04 plasmid | 58 | 309 |
| VP64-R | ATGGGAGAACTCGACTTCTGAGGAGCCGCCAGAGGAGC |  |  |  |
| NLS-TadA 8e-F | GGCTCCTCTGGCGGCTCCCCAAAGAAGAAGCGCAAGGTGTCAGAAGTCGAGTTCTCCCATG |  | 58 | 893 |
| *Sbf*Ⅰ-R | GCCATCTCGTTGGAGAAAATCTCCTGCAGG |  |  |  |
| OsNRT-F | GGCGACTAGATATCTAAACCATTA | Construction of rAKBE plasmid in rice | 95–25 | 24 |
| OsNRT-R | AAACTAATGGTTTAGATATCTAGT |  |  |  |
| OsDEP1-F | GGCGAGACAAGCTTGGCCCTCTTT | Construction of rAKBE plasmid in rice | 95–25 | 24 |
| OsDEP1-R | AAACAAAGAGGGCCAAGCTTGTCT |  |  |  |
| OsWaxy-T1-F | GGCGTTGTAATCAACTCCAGTGTC | Construction of rAKBE plasmid in rice | 95–25 | 24 |
| OsWaxy-T1-R | AAACGACACTGGAGTTGATTACAA |  |  |  |
| OsWaxy-T2-F | GGCGCGCCAAGTACGACGCAACCA | Construction of rAKBE plasmid in rice | 95–25 | 24 |
| OsWaxy-T2-R | AAACTGGTTGCGTCGTACTTGGCG |  |  |  |
| OsWaxy-T3-F | GGCGCCATAAGTTCCTTTGAAGTA | Construction of rAKBE plasmid in rice | 95–25 | 24 |
| OsWaxy-T3-R | AAACTACTTCAAAGGAACTTATGG |  |  |  |
| Hi T-Wx T1-F | GGAGTGAGTACGGTGTGCGCAGTTGGACTGAGCTTAGC | 1st PCR for Hi-TOM sequencing of sgRNA target site | 58 | 208 |
| Hi T-Wx T1-R | GAGTTGGATGCTGGATGGCTCGTGATGCTTCCAACCATC |  |  |  |
| Hi T-Wx T2-F | GGAGTGAGTACGGTGTGCGCATGGACGTCAGCGAGTG | 1st PCR for Hi-TOM sequencing of sgRNA target site | 58 | 267 |
| Hi T-Wx T2-R | GAGTTGGATGCTGGATGGCTCCGCCTGCAACGCC |  |  |  |
| Hi T-Wx T3-F | GGAGTGAGTACGGTGTGCCAGCACTCGAGGCTCCTAG | 1st PCR for Hi-TOM sequencing of sgRNA target site | 58 | 238 |
| Hi T-Wx T3-R | GAGTTGGATGCTGGATGGCAGTGGGCCAGTGTGCC |  |  |  |
| HI T-NRT-F | GGAGTGAGTACGGTGTGCTAGATATACGGTCTCGCAAGCC | 1st PCR for Hi-TOM sequencing of sgRNA target site | 58 | 220 |
| HI T-NRT-R | GAGTTGGATGCTGGATGGGGGCATGTGTATGTCGACTCTAG |  |  |  |
| Hi T-OsDEP1-F | GGAGTGAGTACGGTGTGCGTACTGTTTCTAGGCGGAGGTG | 1st PCR for Hi-TOM sequencing of sgRNA target site | 58 | 207 |
| Hi T-OsDEP1-R | GAGTTGGATGCTGGATGGGGCCACTCAGGTCAGTTGTC |  |  |  |
| OsDEP1-F | GCGAGATCACGTTCCTCAAGG | PCR for Sanger sequencing of *OsDEP1* target site | 58 | 601 |
| OsDEP1-R | CCATGGTGGCAGCCCATAAC |  |  |  |
| OsWaxy 145-F | GCAGACAGGTACGAGAGGGTG | PCR for Sanger sequencing of *OsWaxy*-T1 target site | 58 | 770 |
| OsWaxy 275-R | GAAAGCGAAACGGCCCTGG |  |  |  |
| OsWaxy 170-F | GCAGTTGGACTGAGCTTAGCT | PCR for Sanger sequencing of *OsWaxy*-T3 target site | 58 | 430 |
| OsWaxy 235-R | CCAGTGGGCCAGTGTGCC |  |  |  |
| OsWaxy 270-F | TCCTACCAGGGCCGTTTCGC | PCR for Sanger sequencing of *OsWaxy*-T2 target site | 58 | 613 |
| OsWaxy 390-R | CCTCCGCCTGCAACGCCT |  |  |  |
| OsNRT1.1B-F | CGAGATGGAGCGAGTATATTCAGG | PCR for Sanger sequencing of *OsNRT1.1B* target site | 58 | 536 |
| OsNRT1.1B-R | GTCACTATGTGGAGGAGGAGGTG |  |  |  |
| DEP1-OT-F1 | AAGCTGTGGACTGTTTGGGG | PCR for Sanger sequencing of *OsDEP1* off target site | 58 | 460 |
| DEP1-OT-R1 | TAGGGATGGCGGCATCTAGAC |  |  |  |
| DEP1-OT-F2 | GTTTGCGGTTGTGATGACCCA |  | 58 | 395 |
| DEP1-OT-R2 | CCTCTTCTCCTCGATTCGGC |  |  |  |
| NRT1.1B-OT-F1 | ACTCCAGAGGACACCGTCTT | PCR for Sanger sequencing of *OsNRT1.1B* off target site | 58 | 596 |
| NRT1.1B-OT-R1 | AAGTTCACCTGCCATCCCTTAT |  |  |  |
| NRT1.1B-OT-F2 | CTGGCTCAAAACTGGCTTGC |  | 58 | 544 |
| NRT1.1B-OT-R2 | ACCAGTGGCACTTTTCACCATC |  |  |  |
| Waxy-T1 OT-F1 | TCTCCTCCTCCTCCTCCTCT | PCR for Sanger sequencing of *OsWaxy*-T1 off target site | 58 | 522 |
| Waxy-T1 OT-R1 | TTGCCAAGCAAAACGAGCAAT |  |  |  |
| Waxy-T1 OT-F2 | AAACGGAGGGAGTACAGTGG |  | 58 | 576 |
| Waxy-T1 OT-R2 | GCCATGCGAGCTTGAATGTT |  |  |  |
| Waxy-T1 OT-F3 | CGTTCTTCCCAGTTGACCGAG |  | 58 | 547 |
| Waxy-T1 OT-R3 | GCTGTGGTATCCCAAGGTCCG |  |  |  |
| Waxy-T2 OT-F1 | TTCCTGCCACTGTTTTCCAATCAT | PCR for Sanger sequencing of *OsWaxy*-T2 off target site | 58 | 520 |
| Waxy-T2 OT-R1 | TCTGAATCTTCACCGGTGCC |  |  |  |
| Waxy-T2 OT-F2 | AGACACACTTTGGGCCCAAAAC |  | 58 | 559 |
| Waxy-T2 OT-R2 | GTAGTTGATCTGCTCGCCGTTG |  |  |  |
| Waxy-T2 OT-F3 | GTAGTTGATCTGCTCGCCGTT |  | 58 | 559 |
| Waxy-T2 OT-R3 | AGACACACTTTGGGCCCAA |  |  |  |
| Waxy-T3 OT-F1 | TGGGACTTTTCAGTTCGAAGACTTTTG | PCR for Sanger sequencing of *OsWaxy*-T3 off target site | 58 | 593 |
| Waxy-T3 OT-R1 | CCCACTATACCACGTGAGGCA |  |  |  |
| Waxy-T3 OT-F2 | CGTGTCTTTGTTGCTGTCGGG |  | 58 | 543 |
| Waxy-T3 OT-R2 | CCCTGTCAATAGCTAGCTCCCA |  |  |  |
| Waxy-T3 OT-F3 | ATTCTCTGCCAACCGCTAGC |  | 58 | 534 |
| Waxy-T3 OT-R3 | AGCCTAGGACCAGCACTGTACT |  |  |  |
| OsDEP1-qF | GCGACGAGCCATGCTGTAAG | RT-PCR for detecting expression levels of *OsDEP1* | 58 | 150 |
| OsDEP1-qR | AGCTTGGACAGGAGCACGAG |  |  |  |
| OsNRT-qF | ACAAGGTCACCGGCCATGG | RT-PCR for detecting expression levels of *OsNRT1.1B* | 58 | 162 |
| OsNRT-qR | GGCGCTTCTCCTTGTAGACG |  |  |  |

**Table S2. The sequences of targets used in this study**

| **Targeted loci** | **Name and sequences of targets** | **Targeted loci in the genome (bp)** |
| --- | --- | --- |
| *OsDEP1* | AGACAAGCTTGGCCCTCTTTGGG | Intron 1: 456-478 |
| *OsNRT1.1B* | ACTAGATATCTAAACCATTAAGG | Intron 1: 1538-1560 |
| *OsWaxy*-T1 | CCTGACACTGGAGTTGATTACAA | Exon 4: 869-891 |
| *OsWaxy*-T2 | CGCCAAGTACGACGCAACCACGG | Exon 8: 1825-1847 |
| *OsWaxy*-T3 | CCATACTTCAAAGGAACTTATGG | Exon 5: 1067-1089 |

Note: PAM sequence is underlined in each target.

**Table S3. The A-to-G base editing efficacies of different rAKBEs at different targeted loci in rice protoplast**

| **Targets** | **rAKBEs** | **A-to-G** | | |
| --- | --- | --- | --- | --- |
|  |  | **Highest efficiency (%)** | **Lowest efficiency (%)** | **Average efficiency (%)** |
| *OsDEP1* | ABE8e | 8.91 | 5.01 | 6.67±2.02 |
|  | ABE8e-VP64 | 10.81 | 9.45 | 10.11±2.02 |
|  | rAKBE01 | 8.99 | 6.65 | 7.51±1.28 |
|  | rAKBE02 | 10.20 | 6.47 | 8.31±1.87 |
|  | rAKBE03 | 14.38 | 13.28 | 13.65±0.63 |
|  | rAKBE04 | 16.63 | 13.60 | 15.42±1.60 |
| *OsNRT1.1B* | ABE8e | 10.60 | 8.63 | 9.49±1.01 |
|  | ABE8e-VP64 | 14.84 | 11.53 | 12.83±1.77 |
|  | rAKBE01 | 8.37 | 7.18 | 7.84±0.61 |
|  | rAKBE02 | 10.99 | 10.40 | 10.76±0.31 |
|  | rAKBE03 | 14.18 | 12.42 | 13.49±0.94 |
|  | rAKBE04 | 17.87 | 14.35 | 16.52±1.90 |
| *OsWaxy-*T1 | ABE8e | 8.65 | 6.82 | 7.50±1.00 |
|  | ABE8e-VP64 | 10.14 | 6.87 | 8.68±1.66 |
|  | rAKBE01 | 7.87 | 6.29 | 7.00±0.80 |
|  | rAKBE02 | 11.08 | 8.55 | 9.91±1.27 |
|  | rAKBE03 | 16.07 | 12.38 | 14.38±1.86 |
|  | rAKBE04 | 16.58 | 14.30 | 16.52±1.90 |
| *OsWaxy*-T2 | ABE8e | 9.67 | 7.71 | 8.64±0.98 |
|  | ABE8e-VP64 | 13.65 | 9.07 | 11.13±2.33 |
|  | rAKBE01 | 8.73 | 7.40 | 8.15±0.68 |
|  | rAKBE02 | 10.66 | 7.28 | 9.37±1.83 |
|  | rAKBE03 | 12.37 | 9.35 | 10.58±1.59 |
|  | rAKBE04 | 14.24 | 11.50 | 13.19±1.48 |
| *OsWaxy*-T3 | ABE8e | 6.33 | 4.41 | 5.44±0.97 |
|  | ABE8e-VP64 | 9.33 | 4.23 | 6.89±2.56 |
|  | rAKBE01 | 8.23 | 6.68 | 7.50±0.78 |
|  | rAKBE02 | 8.21 | 7.45 | 7.89±0.40 |
|  | rAKBE03 | 13.18 | 10.27 | 11.72±1.02 |
|  | rAKBE04 | 15.55 | 10.33 | 13.31±2.69 |

**Table S4. The A-to-Y base editing efficacies of different rAKBEs at different targeted loci in rice protoplast**

| **Target** | **rAKBEs** | **A-to-C efficiency (%)** | | | **A-to-T efficiency (%)** | | | **A-to-Y efficiency (%)** | | |
| --- | --- | --- | --- | --- | --- | --- | --- | --- | --- | --- |
|  |  | **Highest efficiency** | **lowest efficiency** | **Average efficiency** | **Highest efficiency** | **Lowest efficiency** | **Average efficiency** | **Highest efficiency** | **Lowest efficiency** | **Average efficiency** |
| *OsDEP1* | rAKBE01 | 0.00 | 0.00 | 0.00 | 0.00 | 0.00 | 0.00 | 0.00 | 0.00 | 0.00 |
|  | rAKBE02 | 0.64 | 0.41 | 0.53±0.16 | 1.22 | 0.72 | 0.97±0.35 | 1.86 | 0.41 | 0.99±0.76 |
|  | rAKBE03 | 0.99 | 0.80 | 0.89±0.10 | 1.56 | 1.41 | 1.48±0.08 | 2.40 | 2.33 | 2.36±0.04 |
|  | rAKBE04 | 1.59 | 1.04 | 1.30±0.28 | 2.21 | 1.74 | 1.97±0.24 | 3.48 | 2.99 | 3.27±0.25 |
| *OsNRT1.1B* | rAKBE01 | 0.54 | 0.35 | 0.47±0.11 | 0.00 | 0.00 | 0.00 | 0.54 | 0.35 | 0.47±0.11 |
|  | rAKBE02 | 1.12 | 0.70 | 0.91±0.21 | 0.97 | 0.71 | 0.85±0.13 | 2.09 | 1.57 | 1.76±0.29 |
|  | rAKBE03 | 1.63 | 0.98 | 1.23±0.35 | 1.46 | 1.38 | 1.41±0.04 | 3.01 | 2.38 | 2.64±0.33 |
|  | rAKBE04 | 1.96 | 1.04 | 1.65±0.53 | 2.66 | 1.47 | 2.22±0.65 | 4.62 | 2.51 | 3.86±1.17 |
| *OsWaxy*-T1 | rAKBE01 | 0.00 | 0.00 | 0.00 | 0.00 | 0.00 | 0.00 | 0.00 | 0.00 | 0.00 |
|  | rAKBE02 | 0.00 | 0.00 | 0.00 | 0.81 | 0.64 | 0.72±0.09 | 0.81 | 0.64 | 0.72±0.09 |
|  | rAKBE03 | 1.24 | 0.93 | 1.08±0.16 | 1.52 | 1.36 | 1.45±0.08 | 2.60 | 2.45 | 2.53±0.08 |
|  | rAKBE04 | 1.60 | 1.13 | 1.33±0.24 | 2.95 | 1.90 | 2.51±0.54 | 4.55 | 3.16 | 3.84±0.70 |
| *OsWaxy*-T2 | rAKBE01 | 0.00 | 0.00 | 0.00 | 0.00 | 0.00 | 0.00 | 0.00 | 0.00 | 0.00 |
|  | rAKBE02 | 0.00 | 0.00 | 0.00 | 1.68 | 0.43 | 1.21±0.68 | 1.68 | 0.43 | 1.21±0.68 |
|  | rAKBE03 | 1.33 | 0.97 | 1.12±0.19 | 2.77 | 1.58 | 1.99±0.67 | 3.82 | 2.55 | 3.11±0.65 |
|  | rAKBE04 | 1.36 | 1.16 | 1.24±0.11 | 2.43 | 1.41 | 1.85±0.52 | 3.62 | 2.57 | 3.09±0.53 |
| *OsWaxy*-T3 | rAKBE01 | 0.00 | 0.00 | 0.00 | 0.00 | 0.00 | 0.00 | 0.00 | 0.00 | 0.00 |
|  | rAKBE02 | 0.00 | 0.00 | 0.00 | 0.60 | 0.48 | 0.54±0.08 | 0.60 | 0.48 | 0.54±0.08 |
|  | rAKBE03 | 1.28 | 0.80 | 1.07±0.25 | 1.83 | 1.23 | 1.54±0.30 | 2.96 | 2.03 | 2.61±0.50 |
|  | rAKBE04 | 1.52 | 1.26 | 1.41±0.13 | 2.42 | 1.72 | 2.03±0.36 | 3.94 | 2.98 | 3.44±0.48 |

**Table S5. The small indels efficacies of different rAKBEs at different targeted loci in rice protoplast**

| **Targets** | **rAKBEs** | **small indels** | | |
| --- | --- | --- | --- | --- |
|  |  | **Highest efficiency (%)** | **Lowest efficiency (%)** | **Average efficiency (%)** |
| *OsDEP1* | rAKBE01 | 0.00 | 0.00 | 0.00 |
|  | rAKBE02 | 0.00 | 0.00 | 0.00 |
|  | rAKBE03 | 0.00 | 0.00 | 0.00 |
|  | rAKBE04 | 1.39 | 0.74 | 1.03±0.33 |
| *OsNRT1.1B* | rAKBE01 | 0.00 | 0.00 | 0.00 |
|  | rAKBE02 | 0.00 | 0.00 | 0.00 |
|  | rAKBE03 | 0.92 | 0.52 | 0.72±0.20 |
|  | rAKBE04 | 1.09 | 0.83 | 0.98±0.14 |
| *OsWaxy-*T1 | rAKBE01 | 0.00 | 0.00 | 0.00 |
|  | rAKBE02 | 0.00 | 0.00 | 0.00 |
|  | rAKBE03 | 0.75 | 0.41 | 0.56±0.17 |
|  | rAKBE04 | 1.05 | 0.58 | 0.77±0.25 |
| *OsWaxy*-T2 | rAKBE01 | 0.00 | 0.00 | 0.00 |
|  | rAKBE02 | 0.00 | 0.00 | 0.00 |
|  | rAKBE03 | 0.00 | 0.00 | 0.00 |
|  | rAKBE04 | 0.60 | 0.50 | 0.56±0.06 |
| *OsWaxy*-T3 | rAKBE01 | 0.00 | 0.00 | 0.00 |
|  | rAKBE02 | 0.00 | 0.00 | 0.00 |
|  | rAKBE03 | 0.00 | 0.00 | 0.00 |
|  | rAKBE04 | 0.95 | 0.39 | 0.58±0.32 |

**Table S6. Analysis of potential off-target effects**

| **Target** | **Name of putative off-target site** | **Putative off-target locus** | **Sequence of the putative off-target site** | **No. of mismatching bases** | **No. of individual plants sequenced** | **No. of plants with mutations** |
| --- | --- | --- | --- | --- | --- | --- |
| *OsDEP1*  sgRNA | OFF1 | Chr06:20054044 | ACACAAATTTGGCCCTGTTT GGG | 4 | 148 | 0 |
|  | OFF2 | Chr07:7389520 | CGACAAGCTTGGATCTGTTT GGG | 4 | 148 | 0 |
| *OsNRT1.1B*  sgRNA | OFF1 | Chr01:18621557 | ACTAGATATCTATACTACTA TGG | 3 | 99 | 0 |
|  | OFF2 | Chr03:11117140 | ACTAGATTTCTAAATGATTA TGG | 3 | 99 | 0 |
| *OsWaxy*-T1  sgRNA | OFF1 | Chr06:6167859 | TAGTAATGAACTCTAATGTC AGG | 4 | 107 | 0 |
|  | OFF2 | Chr11:15226886 | TAGTAATCACATCCAGTGTC TGG | 3 | 107 | 0 |
|  | OFF3 | Chr07:7943744 | TTGTAGACATCTCCAGTGAC AGG | 4 | 107 | 0 |
| *OsWaxy*- T2  sgRNA | OFF1 | Chr06:10184414 | CGCCAAGGACGACGCGACGA CGG | 3 |  | 0 |
|  | OFF2 | Chr11:4656829 | CGGCAAGCACAACGCAATCA CGG | 4 | 103 | 0 |
|  | OFF3 | Chr11:4702191 | CGGCAAGCACAACGCAATCA CGG | 4 | 103 | 0 |
| *OsWaxy*-T3  sgRNA | OFF1 | Chr02:12684999 | CAATAAGTAACTTTGAAATA TGG | 4 | 91 | 0 |
|  | OFF2 | Chr11:12670097 | TCATAATTTCCTTTGAAATA AGG | 4 | 91 | 0 |
|  | OFF3 | Chr10:171419 | TCATAATTTCCTTTGAAATA AGG | 4 | 91 | 0 |

Note: The PAM motif is underlined; mismatching bases are shown in red.
